# Application of guar (*Cyamopsis tetragonoloba* L.) gum in food technologies: A review of properties and mechanisms of action

**DOI:** 10.1002/fsn3.3383

**Published:** 2023-05-07

**Authors:** Sima Tahmouzi, Heidar Meftahizadeh, Saba Eyshi, Amin Mahmoudzadeh, Behnam Alizadeh, Neda Mollakhalili‐Meybodi, Mehrnaz Hatami

**Affiliations:** ^1^ Department of Food Sciences and Technology School of Public Health Shahid Sadoughi University of Medical Sciences Yazd Iran; ^2^ Department of Nature Engineering Faculty of Agriculture & Natural Resources Ardakan University Ardakan Iran; ^3^ Department of Food Sciences and Technology School of Nutrition and Food Sciences Tabriz University of Medical Sciences Tabriz Iran; ^4^ Department of Food Science and Technology Faculty of Agriculture University of Tabriz Tabriz Iran; ^5^ Department of Medicinal Plants Faculty of Agriculture and Natural Resources Arak University Arak Iran

**Keywords:** additive, application, guar gum, mechanism

## Abstract

With the world continuing to push toward modernization and the consumption of processed foods growing at an exponential rate, the demand for texturizing agents and natural additives has also risen as a result. It has become increasingly common to use thickening agents in food products to modify their rheological and textural properties and enhance their quality characteristics. They can be divided into (1) animal derived (chitosan and isinglass), (2) fermentation produced (xanthan and curdlan), (3) plant fragments (pectin and cellulose), (4) seaweed extracts (agar and alginate), and (5) seed flours (guar gum and locust bean gum). The primary functions of these materials are to improve moisture binding capacity, modify structural properties, and alter flow behavior. In addition, some have another responsibility in the food sector, such as the main ingredient in the delivery systems (encapsulation) and nanocomposites. A galactomannan polysaccharide extracted from guar beans (*Cyamopsis tetragonolobus*), known as guar gum (GG), is one of them, which has a wide range of utilities and possesses popularity among scientists and consumers. In the world of modernization, GG has found its way into numerous industries for use in food, cosmetics, pharmaceuticals, textiles, and explosives. Due to its ability to form hydrogen bonds with water molecules, it imparts significant thickening, gelling, and binding properties to the solution as well as increases its viscosity. Therefore, this study is aimed to investigate the characteristics, mechanisms, and applications of GG in different food technologies.

## Graphical abstract

A galactomannan polysaccharide extracted from guar beans, known as guar gum (GG), is one of them, which has a wide range of utilities and possesses popularity among scientists and consumers. This study was aimed to investigate the characteristics, mechanisms, and applications of GG in different food technologies.

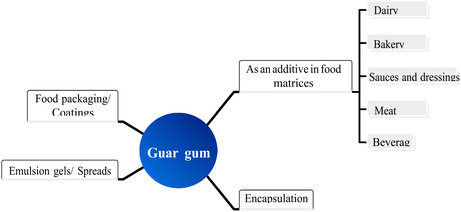

## 1 INTRODUCTION

There are several different cultivars of guar bean (*Cyamopsis tetragonoloba* L.) plant, cultivated primarily in India and Pakistan, as well as in the United States to a lesser extent (Mudgil et al., 2014). In plants, carbohydrates produced by photosynthesis are well known for their essential role as rich sources of energy and carbon skeletons for organic compounds and storage components (Zheng et al., 2023). The GG is a polysaccharide soluble in cold water and derives from the endosperm of the guar plant by extracting its endosperm (Prem et al., 2005). There are mainly three types of polysaccharides in the GG, with a molecular weight range from 50,000 to 8,000,000 Da (Mahmoud, 2000). It is made up mainly of polysaccharides with a high molecular weight (Kawamura, 2008). There is a significant similarity between guar gum and locust bean gum because they both contain the complex carbohydrate polymer galactose and mannose with different ratios of the two sugars. GG is composed of a 2:1 ratio of mannose to galactose (M/G) (Kulicke et al., 1996). There are several types of galactomannans, but in general, they consist of mannose chains linked together by galactose residues or side chains on either end. Accordingly, when it comes to GG, there is one galactose side chain for every two mannose units (Roberts, 2011).

Guar gum can be used for a variety of industrial purposes due to its ability to form hydrogen bonds with water molecules (Tood et al., 1990). Also, it has an excellent water‐thickening ability, eight times greater than other agents (e.g., other gums (Arabic and Tragacanth) or cornstarch), and only a small quantity is needed to achieve sufficient viscosity (Yousif et al., 2017). As a result, fewer requirements reduce costs, which is economical. It is widely used in the food (Gupta & Variyar, 2018), paper, pharmaceuticals, cosmetics (Madni et al., 2021), oil well drilling, explosive, and textile industries (Sharma, 2005) in the form of guar gum powder. It is primarily used as a thickener and stabilizer in food matrices and has recently been used as a film‐making material and a delivery system component. However, native gums are often found lacking in certain functional properties. As a result, the gum is chemically modified to meet the requirements of specific applications (Prabhanjan et al., 1989). GG may be modified in several ways, including oxidation, depolymerization, carboxymethylation, hydroxyalkylation, sulphation, and quaternization (Gupta & Verma, 2014). In this regard, Various grades and qualities of guar gum derivatives are classified according to their modification method, particle size, viscosity at a given concentration, and their rate of development. Some of them are carboxymethyl guar gum (Dodi et al., 2011), hydroxymethyl guar gum (Lapasin et al., 1991), hydroxypropyl guar gum (HPG) (Lapasin et al., 1995), O‐2hydroxy‐3‐ (trimethylammonia propyl), guar gum (HTPG), O‐carboxymethyl‐O‐hydroxypropyl guar gum (CMHPG) (Shi & Zhang, 2007), methylated guar gum, partially hydrolyzed guar gum (PHGG), sulfated guar gum (Kazachenko et al., 2020), and guar gum esters (Dong & Tian, 1999). Over the past few decades, a wide range of applications has been developed for hydroxyalkyl derivatives, both in industry and food (as thickener and emulsifier) (Kazachenko et al., 2022). According to the study by Zhang et al. (2005), the aqueous CMHPG solution investigated had the greatest viscous flow activation energy among HTPG, HPG, and PHGG (Zhang et al., 2005). Considering its high viscosity, guar gum is used in food products at a ratio of 1/100 g/g (1%) (Flammang et al., 2006). Consequently, it can be processed into a product called partially hydrolyzed guar gum (PHGG) to reduce its viscosity (Bachate et al., 2021). Because of this, choosing the type of gum derivative and the modification method is crucial when considering the environment in which guar gum will be used.

A common application is in dairy products, where it thickens yogurt (Khairi et al., 2020), liquid cheese products, and kefir, as well as helps maintain ice cream homogeneity and texture (Javidi et al., 2016). Pastry fillings prevent the “weeping” of water from the filling, making the crust crisp and increasing dough yield and texture in baked goods (Ribotta et al., 2004; Salehi, 2019). It is used as a binder and fat replacement in the case of meat and meat products (Ulu, 2006). In addition, it provides added stability, texture, and appearance to salad dressings, sauces (such as ketchup, mustard, and mayonnaise), relishes, and other prepared foods (Wang et al., 2016). Moreover, it is beneficial in the control of some health problems, like diabetes. While guar gum does not necessarily provide essential nutrients, it is low in calories and high in fiber, which helps to give a satiated feeling (Srinivasan, 2020). As a soluble‐fiber source in food products, guar gum can also be used as a source of dietary fiber and is safe at a daily usage of 20 g (Flammang et al., 2006; Grabitske & Slavin, 2009). For instance, it could be used instead of cocoa butter to create low‐fat chocolate, benefiting people with diabetes (Amir et al., 2013).

There is no doubt that developing biodegradable coatings and nanocomposite films to improve the shelf life and quality of perishable food products (e.g., minimally processed fresh produce) (Bourtoom, 2008; Krochta, 2017) and enhance the digestion of foods by modifying their dispersibility status and enhancing their bioavailability are a critical topic over the world (Marcillo‐Parra et al., 2021). In this regard, gums, especially GG, are a good choice for improving the sustainability of biodegradable packaging due to their polymeric structure and accessibility (Thombare et al., 2016), and they are also able to hold insoluble particulates (flavorings, drugs, and other active ingredients) (Saifullah et al., 2019). This is why both consumers and manufacturers prefer to use guar gum or PHGG in different fields and food matrices. The purpose of this review article is to provide an overview of the composition, characteristics, food applications, and health benefits of guar gum.

## 2 GENERAL PROPERTIES

With the increasing awareness of consumers about the safety and health aspects of the food sector, researchers and food industry manufacturers are trying to provide safe and high‐quality products. In addition to satisfying nutritional needs, food products should also meet the sensory needs of consumers. The defects and problems of food processing were also solved by using new methods and different natural additives, and since the demand is to consume natural food, the use of chemical additives in food processing is decreasing. Hydrocolloids are long‐chain compounds that easily disperse, are swollen in water, and dissolve completely or partially. They change the physical properties of the solution into a gel form and are capable of thickening, emulsifying, coating, and stabilizing (Li & Nie, 2016). The functional properties of hydrocolloids are significantly dependent on their physicochemical properties, including molecular weight, chemical compounds, monosaccharide sequence, structure, glycosidic bond position, particle size, viscosity, etc. (Fathi et al., 2016; Zeece, 2020).

### 2.1 Physical properties

**FIGURE 1:**
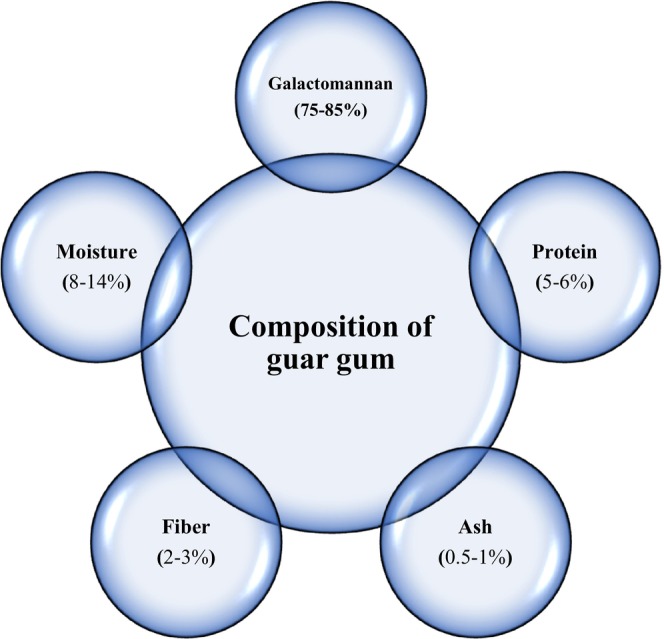
Composition of typical guar gum.

GG is obtained from the endosperm of the guar seed *Cyamopsis tetragonolobus* and *Cyamopsis psoraloides*, family *Leguminosae*, which was separated from the hull and germ and then ground into different particle sizes (Bogdanova Popov et al., 2017; Dehghani Soltani et al., 2021) Endosperm, germ, and hull comprise 45%, 40%, and 15% of the seed, respectively (Feiner, 2006; Maier et al., 1993). To obtain endosperm from guar seeds, the seeds must be entered into two‐level mills. The seeds coming out of the mill still have the hull and germ, so the endosperm is heated to soften the shell, and the endosperm is re‐entered into the mill to remove the hull and the germ completely. Next, the endosperm is powdered. The outer hull and germ, which is a meal, are used as animal feed (Kapoor et al., 2013). This gum is white and light grayish. Guar is a high‐weight polymer soluble in water; its weight is reported to be 22,000 Daltons (Feiner, 2006, Maier et al., 1993). The thermal resistance of guar gum is between 80 and 95°C (Feiner, 2006, Maier et al., 1993). The components of guar are shown in Figure 1. Guar is widely used in the food industry, it also emulsifies, and bind water to prevent ice crystal in a frozen product and postpone many liquid‐solid systems. Also, various types of research have shown that this gum reduces blood cholesterol and controls obesity and type 2 diabetes (; Dehghani Soltani et al., 2021).

#### 2.1.1 Solubility

Guar gum dissolves in polar solutions such as water, hydrazine, formamide, ethylene diamine, and liquid ammonia. Various factors, such as increasing temperature and decreasing pH and particle size, increase the solubility of guar in water, while the presence of salt and sugar decreases solubility (Maier et al., 1993). The rate of hydration depends on the concentration of galactomannan; therefore, the concentration (0.5%–1.2% w/v) of the hydration rate accelerated with the increase in the concentration of galactomannan, but at concentrations higher than 1.2% w/v, the rate of hydration also decreased; particle size and its distribution are important factors in guar hydration rate (Wang et al., 2003).

#### 2.1.2 Rheology

The rheological properties of hydrocolloids provide the basis for their wide application in industry. The unique rheological behavior of hydrocolloids can be attributed to the presence of a large number of hydroxyl groups in their structure, which leads to the formation of hydrogen bonds in aqueous systems (Kapoor et al., 2013). Guar gum is a non‐Newtonian material and, in an aqueous solution, shows pseudoplastic behavior, which means that the viscosity decreases due to the shear rate. Studies have also shown that in 1% guar solution, the module is the surmount loss modulus over storage modulus at low frequencies but at higher frequencies storage modulus dominates the loss modulus (Bogdanova Popov et al., 2017; Mudgil et al., 2014).

#### 2.1.3 Viscosity

The viscosity of a substance is the internal resistance of its different parts against jerking and displacement, and this quality depends on the molecular dimensions of the dissolved solids in the system. Studying the viscosity of a solution can provide useful information about the size, shape, and distance of molecules. The resistance between the different layers of a liquid or semi‐liquid system in the flow is obtained due to the Brownian motion of the molecules in the inner layers. This important physical property depends on various factors, such as the size and number of macromolecules in the fluid structure. Gums have the ability to make products with high viscosity in low concentration and hence are used in various food industries. Gums are classified into two categories: linear and branched. Linear glues occupy more space and, as a result, are more viscous than branched glues and tend to form films quickly, while branched glues form gels more efficiently and have greater solubility in water. Guar gum is structurally a polysaccharide, which is a long chain with very short branches; hence, it can have the behavior of linear and branched gums. Therefore, this gum quickly absorbs water and makes a very thick solution (Srichamroen, 2013). GG shows its thixotropic nature because it turns into a liquid with continuous stirring, and it has been determined that a high concentration of 1% of GG in an aqueous solution increases the thixotropic behavior (Wang et al., 2016). Like other gums, the viscous solution formed by guar gum depends on factors such as molecular weight, degree of branching (Ma & Boye, 2013), time, temperature, concentration, ionization, and pH. Increasing the concentration of guar increases the viscosity of the solution (Maier et al., 1993) due to the increased interaction between molecular chains of guar and water (Mudgil et al., 2014). In addition, it has been determined that a concentration greater than 0.045 g/dL of guar gum will lead to an increase in viscosity (Yan et al., 2005). Increasing temperature accelerates the solubility of gum. However, high temperatures cause its decomposition because it destroys the order of water around the guar or decreases the intermolecular interactions; due to thermal degradation, viscosity decreases with increasing temperature (121°C) (Kök et al., 1999). Although the maximum viscosity of guar gum is obtained at a temperature of 25–40°C. Guar solution with a concentration of 0.5% shows the highest viscosity at a temperature of 25°C and a constant temperature; the 0.5% solution acts like a Newtonian system (Srichamroen, 2013). The viscosity decreased with the increase of time, which is probably due to enzymatic degradation (Mudgil et al., 2014).

#### 2.1.4 Gelation

One of the most important properties of guar is gel formation. The gel is an intermediary between solid and liquid, which has both solid (elastic) and liquid (flow) properties. Gelation is a phenomenon that involves the cross‐linking of polymer chains to form a three‐dimensional network that traps water within it to form a rigid structure that resists flow stresses under pressure and maintains its structure. Hydrocolloids form gels through hydrogen bonds, cation‐based cross‐links, and hydrophobic bonds. The gel formed by guar depends on factors such as temperature, pH, and concentration of guar. The optimal pH range for gel formation is 7.5–105. Various compounds, including borate and transition metal ions, increase cross‐links with guar gum and increase gelling power, viscosity, and resistance at high temperatures (Maier et al., 1993).

#### 2.1.5 Thickening

One of the essential characteristics of hydrocolloids is thickening, which has led to their use in various food industries, including sauces, jams, etc. This process occurs due to the interweaving of hydrocolloid polymer chains with a solvent higher than the critical concentration. At critical concentrations, the molecules have less mobility and join together, creating an interwoven network and forming the thickening process. This feature depends on various factors such as polymer type, charge density, environmental conditions (temperature and humidity), and type of food system (Mahmood et al., 2017). Due to its hydroxyl groups, GG tends to form hydrogen bonds with water, which can be used as a stabilizer and thickener.

#### 2.1.6 pH

Guar gum is stable in a wide range of pH due to its neutral structure. The highest water absorption occurs in the 8–9, and the lowest water absorption occurs at a pH >10 and <4. At pH <3, glycosidic structures of guar are destroyed, and the viscosity decreases rapidly (Maier et al., 1993). According to research, the lowest viscosity rate occurs at pH 3.5, while at pH 6 and 9, the highest viscosity rate is observed in GG (Zhang et al., 2005).

#### 2.1.7 Salt and sugar

The rheology of GG is very variable, and the effect of salt and sugar on its rheology depends on various factors such as the type and concentration of gum, solution pH, temperature, and other additives. Sugar has positive and negative impacts on the rheology of GG. The addition of sugar enhances the water‐holding capacity of guar gum by forming many hydrogen bonds, hence facilitating an increase in viscosity and consistency. On the other hand, sugar makes a stronger bond with gurami and increases the resistance of the solution against the flow. However, the concentration and amount of added sugar are very important because with the increase in concentration and amount of sugar, the solubility of guar gum in water decreases, and the viscosity reduces. There are different opinions about the effect of salt on the viscosity of guar; however, it has been shown that a high concentration of salt reduces the solubility of guar and reduces viscosity, while a low concentration of salt increases the solubility of guar and increases intra‐ and intermolecular connections; as a result, the viscosity increases (Mudgil et al., 2014). The viscosity of 0.25 guar dissolved in carb bicarbonate treatment was lower than that of water solution, and this reduction in viscosity is due to the reduction in access to water by guar gum and lack of network formation. The added salt reduces the hydration rate of GG. However, the results showed that in the 0.5 treatment, the viscosity of the bicarbonate solution was higher than that of the water solution because the presence of salt changes the charge density and facilitates the addition of GG. By distorting intra‐ and intermolecular communication, salt leads to the expansion of the chain structure and thus increases viscosity (Srichamroen, 2013).

#### 2.1.8 Synergistic effect

Guar gum has a unique structure that consists of linear chains of galactose and mannose with branching points at regular intervals, and hence the synergistic effect of guar gum increases its functional properties when used in combination with other substances such as other hydrocolloids, protein, salt, and sugars. Examples of the synergistic effect of guar gum have been investigated in various studies, including the synergistic effect of guar in combination with xanthan gum. The combination of guar gum and xanthan gum creates a stronger, more elastic, and more viscous solution than each of them separately. Also, studies have shown that the formed gel is stronger and its resistance to shear forces increases, which can be used in products such as salad dressings, sauces, and drinks (Casas et al., 2000; Pai & Khan, 2002). Other synergistic effects of guar gum include its combination with protein. This combination produces heat‐stable gels and creates thicker and creamier textures in products such as yogurt and ice cream (Brennan & Tudorica, 2008; Lee & Chang, 2016; Salih et al., 2020). However, various variables such as the amount of gum, the amount of ingredients used in combination with gum, and the characteristics of the food product are important in the application of the synergistic effect. In a study, it was observed that the combination of xanthan and guar gums is not stable at room temperature, and phase separation occurs after one or two rounds, but when they are kept at 4°C, the system is stable probably because at low‐temperature Brownian motion of molecules is limited and the composition remains stable (Casas et al., 2000). Various studies have shown the synergistic effect of starch with different gums, including guar gum, which led to the optimization of the formulation to obtain the desired properties. It has also been shown that the use of non‐ionic gums such as guar has a synergistic effect with starch and affects the gelatinization and retrogradation behavior of wheat starch; therefore, with increasing temperature, galactomannans interact with amylopectin and lead to an increase in paste viscosity; and the lower the side chains of the galactose, the higher the viscosity of the paste (Funami et al., 2005). GG in different concentrations (%0, 0.3, and 0.6 w/w) at 25°C had an effect on the rheological properties of sweet potato starch paste and therefore it caused an increase in sheet thinning flow with yield stress. Also, with the increase in guar concentration, the apparent viscosity increased (Choi & Yoo, 2009). In a similar study, it was found that the addition of GG to rice starch increased the pseudoplastic property because there is a mannan backbone with galactose branches in the structure of GG, which prevents the formation of intramolecular hydrogen bonds and hence, the molecule is in an expanded form that can easily combine with starch amylose molecules through non‐covalent hydrogen bonds, thus creating an extended compound. It was also observed in this study that the apparent viscosity and consistency index and yield stress increased with the increase in the concentration of galactomannans. Galactomannans are placed in the continuous phase (amylose) and the volume of this phase is reduced, and by increasing the concentration of galactomannans in the continuous phase, they cause high viscosity. The results showed that the consistency index of rice starch increases due to the high hydration capacity of guar (Yoo et al., 2005).

### 2.2 Chemical properties

**FIGURE 2:**
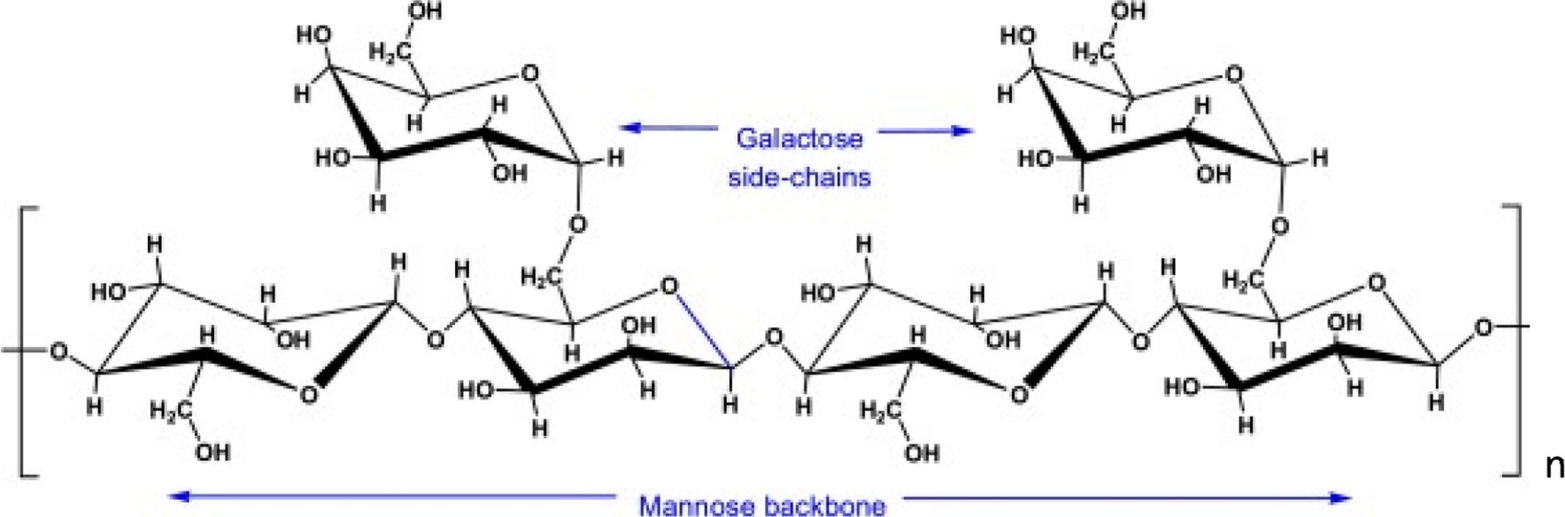
Structure of guar gum.

Guar gum, a galactomannan, is a polymer consisting of two monosaccharide units, mannose and galactose, called galactomannan. The structural units of guar gum include galactopyranosyl and mannopyranosyl. Its structure consists of two D‐mannose units, β1 → 4 linked to the D‐galactose unit α1 → 6 linked to every second mannose unit. The ratio of galactose to D‐mannose is 1:2 (Figure 2) (Srinivasan, 2020). The presence of hydroxyl groups and its tendency to establish hydrogen bonds have used this gum in various industries. The interaction of galactose and mannose units with water molecules increases the viscosity of the solution (Dehghani Soltani et al., 2021). Production of high‐viscosity soluble gum can limit the use of this gum in the food industry so that it can cause molecular weight loss, molecular chain, and viscosity by various methods, including heat, alkali, ultrasonic, acid, and enzyme. In the meantime, acid and enzyme methods are used because of their simplicity and the acquisition of guar with any molecular weight (Li et al., 2018).

## 3 MECHANISMS OF FUNCTION

In meat, gum improves texture and adhesion by creating a gel and prevents syneresis. Dissolving the solubility of the gum in cold water and the swelling and adding it to the meat maintains sensory properties and prevents microbial contamination. Gums are added to mayonnaise as an oil substitute for various reasons, including stability, suspension of solid particles and spices, improvement of thickening, gelation, stabilization, and preservation of sensory properties. Gums added to the formulation of sauce and mayonnaise should have good stability against low pH so as not to affect their functional properties. Therefore, due to its stability in a wide range of pH, guar gum is suitable for creating a solution with high viscosity at low concentrations, as well as reducing blood cholesterol levels and probiotic properties (Ma & Boye, 2013). Yogurt is a widely used fermented dairy product all over the world, which contains many nutritious compounds. Syneresis is one of the important defects in yogurt and reduces shelf life and general acceptance. Research has shown that adding guar gum (up to a concentration of 2%) increases the safety of the product through its stabilizing properties and prevents the breakdown of fat.

On the other hand, increasing the water‐holding capacity and creating a gel increases the viscosity of yogurt, reduces syneresis, and improves the texture of yogurt (Atik et al., 2020). Adding guar gum to rice bran increases the moisture in the product due to its water retention power. Research has also shown that guar reduces peroxide in products and increases shelf life due to its stabilizing properties and prevention of fat breakdown. Water absorption and viscosity increase, as well as intermolecular hydrogenation between side chains by guar gum, have increased the hardness and breakability of this product (Pourfarzad & Yousefi, 2020). In a study, the effect of guar gum on various properties of green Edam cheese was investigated. Guar gum in concentrations of 0.0025, 0.01, and 0.005 w/v was added to low‐fat milk, and cheese was produced. The results showed that adding gum did not have a specific effect on the amount of protein and fat while increasing the concentration of gum, and the amount of moisture decreased due to its interaction with the protein and fat polysaccharide matrix; hence, the temperature (Tonset, Tpeak, and Tendset) on the treatment containing 0.0025 guar was more than other treatments (Oliveira et al., 2010).

Various researchers have used partially hydrolyzed guar gum PHGG in various food industries. Application of high concentration of guar produces high viscosity and interferes with the sensory and processing properties of the product, PHGG is similar to guar gum in its basic molecular structure. PHGG is obtained by enzymatic hydrolysis, and as a result, the chain length and molecular weight are reduced. PHGG is a rich source of soluble dietary fiber, which reduces free fatty acid serum cholesterol and blood glucose concentration. PHGG has lower viscosity and solubility than guar gum (Mudgil et al., 2012, 2016). Adding PHGG to yogurt decreased the viscosity and water‐holding capacity due to the partial hydrolysis of this gum compared to guar gum (Mudgil et al., 2016; Roberts, 2011). Food with water activity (aw) less than 0.6 is called dried food. Among the advantages of drying, we can mention increasing the shelf life, reducing the volume and weight of the food, and easy transportation and storage (Alp & Bulantekin, 2021). A study found that adding guar gum to noodles made from potato, mung bean, and corn increases the viscosity due to the increased swelling capacity of starch granules and prevents leakage. It was also shown that the treatments containing guar gum significantly reduce the cooking time and cooking losses due to combining guar with amylose and reducing the solubility of starch granules. Also, in terms of the texture of the noodles in the presence of gum, their stiffness and cohesion decreased, but the stickiness increased, which was considered to be due to the delayed swelling of the starch granules and less connection of the granules in the presence of gum (Kaur et al., 2015).

In another study, xanthan, guar, and pectin gums were investigated on the physicochemical and sensory properties of kiwi fruit leather. The kiwi leather was placed at a temperature of 60°C, and the gums were added to the sample in concentrations of 0.2%, 0.5%, and 1%. The results showed that the moisture content and pH of the treatments decreased, while there was no change in the content of phenolic compounds. Also, with the increase in the concentration of the gums, the browning index increased, which helped to increase the antioxidant properties of the treatments. Also, the results showed that the 1% guar treatment had the highest amount of ascorbic acid. Increasing the concentration of gums caused a decrease in brightness (L* values), an increase in a*, and a decrease in yellowness (b*), which is related to the browning index. Regarding texture, the tensile strength index increased with the concentration of gums. The adhesion and chewing indices were shown to be the highest in the concentration of 0.5% and 1% guar, respectively, due to the establishment of hydrogen bonds between the gum and the leather and preventing the deformation of the product. 0.5% guar, xanthan, and pectin treatments had the highest score in terms of sensory properties (Barman et al., 2021). Various research has shown that guar improves the mechanical properties of starch, including texture and rheology. Furthermore, the addition of guar to starch preserves starch granules against various mechanical forces during the process, and guar traps starch granules by forming a stable polymer network and delays the release of amylose. As a result of binding guar with amylopectin, it increases the viscosity in the dough.

Among other applications of the combination of guar with starch, it is possible to mention the preservation of moisture due to the water absorption property of guar, followed by the reduction of syneresis, which improves the process of gelatinization. A study showed that the viscosity of the cold paste increases and the gelatinization temperature decreases with the increase in guar concentration (5% corn starch + 0.10% guar gum, 5% corn starch + 0.20% guar gum). In a similar study, adding guar (0.5% w/v) reduced the pasting temperature of yam starch due to the interaction of hydrocolloids with amylose, which leaked from the granules. In addition, due to the interaction of guar with starch and the immobilization of water, as well as the thickening property of guar, the final viscosity of the product increased.

Frying is one of the oldest methods used to prepare food. Nowadays, this method has become one of the most important methods of raw food processing in homes and industry due to the unique characteristics of fried products, including the favorable mouthfeel (crispy surface and juicy central part), pleasant taste, good texture, and golden color (Akdeniz et al., 2006). In the frying process, physical and chemical changes occur, including starch gelatinization, protein denaturation, water evaporation, and lipid oxidation. One of the main problems related to fried foods is the increased amount of product oil during frying. Due to the high consumption of fat and the increased prevalence of diseases such as obesity, cholesterol, and high blood pressure in industrialized countries, the absorption of fat in fried products has been given great attention from a nutritional point of view. Frying conditions (temperature and time), physicochemical characteristics of food (size, shape, density, porosity, amount of oil and initial moisture in the sample, and surface‐to‐weight ratio), and oil characteristics (type and chemical composition and oil quality) are factors that influence the product. The process (pre‐treatments and conditions of the sample after removing it from the oil) is one of the important factors affecting the amount of oil absorption in the fried samples (Asokapandian et al., 2020; Varela & Fiszman, 2011). One of the most successful ways to reduce oil absorption in fried products is to use food coatings before frying the product. The use of hydrocolloids as edible coatings for fried products has been investigated. Hydrocolloids create a uniform coating on the surface of the product and prevent excessive absorption of oil. Since hydrocolloids are hydrophilic, they make a limited permeability against moisture, are an obstacle to the transfer of oxygen and carbon dioxide, and prevent the oxidation of lipids (Liberty et al., 2019; Porta et al., 2012). Research has shown that using guar as a coating has reduced oil absorption and water removal from the fried product. Also, using guar as a coating reduces the absorption of oil and the exit of water from the fried product due to the high stickiness of this gum and the covering of the holes on the product's surface. In terms of texture, the hardness of the product is higher, and the amount of crispness is lower because the use of guar as a coating increases the stickiness, which affects the texture of the product (Yu et al., 2016). In a similar study, hydrocolloid films were investigated, and a reduction in oil absorption and water release was observed. Also, all treatments were acceptable from a sensory point of view (Izadi et al., 2015).

## 4 APPLICATIONS OF GUAR GUM IN THE FOOD INDUSTRY

**FIGURE 3:**
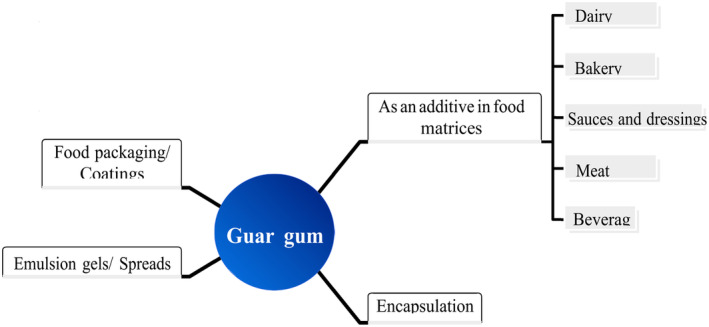
Applications of guar gum in food industry.

Guar gum is primarily used in the food industry (Dehghani Soltani et al., 2021; Herald, 2020). Extensive research has been conducted on GG regarding processing parameters that can be applied directly to the food industry. The molecule chemically consists of a mannose backbone and galactose side chains interspersed throughout (Ellis et al., 2001). In the food industry, it is commonly applied as a food additive with thickening, covering, stabilizing, binding, and suspending many liquid–solid systems because of its solubility in cold water and potential to generate viscous systems even at low concentrations. Also, in some studies, this cheap, widely used gum showed unique properties which make this gum a critical component of food applications, including reduced evaporation rates, modification of rheological properties, improvement of freezing rates, and enhancement of ice crystal formation (von Borries‐Medrano et al., 2016). The industries of dairy, bakery, packaging, and encapsulation are where GG is potentially used (Feiner, 2006). A summary of the various uses of GG is provided in Figure 3. Additionally, it has been rated as “generally recognized as safe (GRAS)” by the US Food and Drug Administration; Code of Federal Regulations Section 184.1339 states the maximum permissible limits in various foodstuffs, which is 0.35% and 2% for bakery products and vegetable juices, respectively. Here are some examples of how GG is used in multiple food industries (Hawthorne, 2005).

### 4.1 Guar gum as an additive in different food matrices

It is well‐known that the corresponding demand for texturizing agents is rising as the world moves toward modernization (Ellis et al., 2001). Indeed, the most important aspects of food products are their textures. Rheological and physicochemical attributes are food quality indicators from a physical and sensory standpoint. Developing a new product, determining its functional properties, and assessing its quality rely strongly on the rheological characterization of its composition (Funami, 2011; Srinivasan, 2020). As a result of human concerns for the quality of life, consumers are also interested in natural products without chemical additives. This gives food companies and scientists a unique chance to target a broad market with products with functional characteristics that possess health‐promoting properties, such as diminishing blood cholesterol levels and nutritional values (Ma & Boye, 2013). Different additives such as traditional materials (starch), a stabilizer obtained from various plants or seeds, and gelatin, an animal by‐product, are used to meet the industry's needs by giving reasonable characteristics to the foodstuffs.

Indeed stabilizers, thickeners, and gelling agents are consumed in different foods to alter rheological properties and texture as well as to enhance quality attributes. It is often called food hydrocolloids when referring to stabilizers, thickeners, and gelling agents. Sea plants, plant seeds, animal connective tissues, and microorganisms are commonly used as natural sources of hydrocolloids. Besides controlling moisture, they include determining the structure, altering flow behavior properties, and providing food products with quality. As a result, they play a crucial role in the acceptance of multiple and significant food functions, including food thickening, structuring, texturizing, and gelling (Gao et al., 2022; Heyman et al., 2010).

Seed gums have been studied separately, combined with microbial polysaccharides or other functional polysaccharides like carrageenan locust bean to improve a sense of mouthfeel, stability, and sensory perceptions of the final product gum and xanthan in recent years (Mahmoud, 2000). Findings have revealed that gums derived from the seeds of the guar plant (*Cyamopsis tetragonoloba*) and, to a lesser extent, from the Tara tree (*Caesalpinia Spinosa*) are widely used in food processing. Approximately 55,000 tons of GG are used annually in food and pet food applications. The most prominent application area of GG is in the food industry. For instance, viscosity and texture are the most commonly used food quality parameters (Yadav et al., 2018). The viscosity of various gums is different; the viscosity of some may be low (Arabic), medium (xanthan), or high (GG) (Yilmazer et al., 1991).

For instance, protein‐based thickeners enhance rheological and nutritional properties. In addition to these cases, gum‐based thickeners can improve physicochemical attributes, digestibility, and rheological characteristics even better than other agents (Mudgil et al., 2017). In this way, one of the critical texturizing aspects of gum‐based thickeners is the ability to stabilize and solidify fluid products. The highly branched structure makes them water soluble. Such gums are able to enhance the viscosity of emulsions by increasing the density of the water (Huang et al., 2001; Ma & Boye, 2013). For example, in gelled milk desserts, even low levels of GG will form a solid milk gel. Several studies suggest that GG acts as a binder in most foods and beverages, thickens liquids, and substitutes fat for a product with a similar profile (Salih et al., 2020). Additional information and different examples are shown in Table 1.

**TABLE 1:** Various guar gum‐based products and their application reason in food industry.

Food industry	Product	Results	Conclusion	References
Bakery goods	Bread	By adding GG, the water absorption capacity of the dough was increased from 60.5% to 68.3%, and the dough stability was increased from 6.5 to 14 min	Sensory acceptance of firmness and volume of baked goods were enhanced	Kohajdová and Karovičová (2008)
	Whole‐meal bread	The results were better when 2% GG was used. Nutritional, textural, and sensorial attributes of samples with GG and pea flour were better than samples with other hydrocolloids	Nutritional, textural, and sensorial attributes of samples with GG and pea flour were better than samples with other hydrocolloids	Mastromatteo et al. (2015)
	Bread	By increasing the oven temperature, all samples got a higher crust temperature, with control samples (48.01%) getting a higher crust temperature than guar gum samples (37.12%)	Results can be related to the high water absorption characteristic of GG	Mohammadi Golchin et al. (2021)
	Gluten‐free bread	The adhesion, porosity, and elongation of bread samples were increased, while the firmness, wrinkle, and specific volume were reduced.	There was an acceptable quality level of the optimal formulation, indicating the potential for industrial use, and to achieve the desired characteristics, 1% GG is recommended for semi‐bulk bread formulations containing potato flour	Moradi et al. (2021)
	Gluten‐free bread and pizza	Using 1% GG (with 38% arabic gum) in GF bread and pizza formulation indicated the acceptability of sorghum‐based bread in order to improve functional properties	Bulk density, emulsifying activity, and oil and water capacity of GG‐based gluten‐free bread samples were boosted	Elkhalifa et al. (2007)
	Pasta	Vendors showed varying levels of increase in dough strength	Improvement of mechanical and rheological properties with GG in durum flour	Sandhu et al. (2015)
	Part‐baked frozen Barbari bread	Fresh bread was found to have increased volume, porosity, color, and firmness with the addition of GG compared to the control sample and xanthan gums. By reducing the air cells and making them more evenly distributed, lipase and amylase contributed to bread's increased porosity. Lipase, with its emulsifying properties, and GG strengthen cell walls and control porosity by maintaining cell walls and preventing them from bursting	In part‐baked bread, bread that has been frozen and re‐baked, the addition of GG in combination with enzymes boosts the crumb consistency	Hejrani et al. (2017)
	Rice cake	Rice cakes containing a mixture of xanthan–guar hydrocolloids without an emulsifier and fat were found to be the firmest due to the thickening of the crumb walls near the air spaces in the rice cakes	Reduction of the staling and improved crumb firmness	Turabi et al. (2008)
	Wheat cake	The addition of 1% GG to the formulation increased the firmness by 58%	The wheat cakes made with guar hydrocolloid were firm	Gómez et al. (2007)
	Biscuit	By adding 0.5% and 1% of GG, the overall sensory and mechanical properties were enhanced	GG reduced the calories in soft dough biscuit formulation	Sudha et al. (2007)
	Gluten‐free bread	GG‐5% flaxseed combination was the most effective in providing a higher specific volume	A softer texture and a better color were shown when the bread baked	Ozkoc and Seyhun (2015)
	Gluten‐free cheese bread	The GF cheese bread made from frozen dough with GG content exceeding 4% had the highest number of pores and the most uniform distribution of pores	The highest level of hardness and chewiness were shown	Zapata et al. (2019)
	Bread	Bread with PHGG had sensory properties similar to the control bread, but the dietary fiber content was significantly higher	GG caused the acceptable sensory and nutrition value in final product	Mudgil (2018)
	Bread	A greater sense of fullness and a decreased desire to eat after subjects were provided with bread containing GG	GG lowered the glycemic index and decreased human insulin production	Ekström et al. (2013)
	Biscuit	GG significantly increased biscuit crispiness and hardness	Overall improvement of sensory properties of biscuits was shown	Singh et al. (2015)
	GF rice cake	Using 1% GG in rice cakes improved crispiness and decreased retrogradation rate by 46% after weeks	GG and xanthan gum inhibit staling in GF rice cakes by decreasing weight loss and retrograde enthalpy, respectively	Sumnu et al. (2010)
	Pasta	A low level of GG (2%–5%) boosted extruder output in pasta and prevented retrogradation through a lubricant effect, improvement of shape definition, and crispness	Functional improvement was achieved by controlling the retrogradation of starch by GG as it is stored during the storage period	Shaikh et al. (2008)
	Pasta	Hardness and texture were improved by 42% and 56%, respectively.	The addition of GG to pasta dough formulation increased the strength of the dough and improved mechanical and rheological properties	Sandhu et al. (2015)
Dairy products	Ice cream	GG added stiffness, slow and uniform meltdown, enhanced whip ability, and prevented shrinkage	Guar‐based ice creams with 0.3% levels of GG were shown satisfactory results in the cohesion and body of the mix	Julien (1953)
	Stirred yogurt	Results showed that body, texture, and creaminess were maintained. Also, it helped to reduce the amount of free water in yogurt by binding it to “water of hydration”	The addition of GG to stirred yogurt dramatically improved the rheological characteristics of the yogurt	Lee and Chang (2016)
	Yogurt	Findings illustrated that PHGG increased the number of live bacteria in yogurt (48%), promoted acid production by lactic acid bacteria (32%), and accelerated curdling by 14%	An acceptable functional and sensory quality could be achieved by incorporating PHGG into yogurt	Mudgil et al. (2016)
	Yogurt	GG (0.2%)–arabic gum (0.5%) mixture significantly increased the viscosity and water‐holding capacity of yogurt and improved the textural quality of yogurt	GG mixture presented the highest and total acceptability scores of yogurts	Rezaei et al. (2011)
	Cheese	GG improved the texture (hardness and stiffness) (32% and 12%) and body of cheese by controlling syneresis through water‐phase management, which regulates weeping from the cheese	GG provided a good texture to cheese as well as other milk products and supported the product to remain consistent in texture and color the product	Gupta and Variyar (2018)
	Ricotta cheese	Due to the interconnected gel‐like structures of the ricotta cheese and the addition of 5% gelatin and 1% GG, the viscoelastic characteristics of the cheese exhibited a shear‐thinning (pseudoplastic) behavior under shear loading. Also, the highest elastic modulus was achieved at a combination of 5% and 0.5% of gelatin and GG, respectively	A gelatin–GG mixture was a better choice to improve the gel strength of the cheese	Hesarinejad et al. (2021)
	Doogh	The results showed significant differences between the sensory and physicochemical properties of the newly formulated doogh due to each factor (storage time, GG%, and Qm%)	This stabilizer maintained homogeneity and presented the highest acceptability	Pirsa et al. (2018)
Meat products	Beef roll	An algin/calcium and a salt/phosphate structure beef roll containing GG have been shown to exhibit an improved water‐holding capacity	GG imparts texture and improves the sensory characteristics of beef roll	Shand et al. (1993)
	Pork sausages	In addition to increasing water‐holding capacity, the hydrocolloid compounds also reduced cooking loss. Hardness, cohesiveness, and chewiness were well maintained compared to the control sausages	Phosphate‐free sausages containing GG were stable for long periods	Park et al. (2008)
	Chicken nuggets	In the sample containing GG, the cooking yields and the stability of emulsions were higher, but the hardness decreased in comparison with the control sample. It has been found that such effects are due to retaining added water before gums were incorporated into the formulation of nugget preparations. Texture properties like springiness were not significantly affected by gum addition.	Sensory features and chemical characteristics are improved with GG in the formulation of chicken nuggets	Yadav et al. (2013)
	Meatball	There was a significant improvement in the cooking yield and fat retention parameters of meatballs when the fat level was reduced from 25% to 10% by adding GG	GG provided a good texture to meatballs	Ulu (2006)
	Meatball	Raw and cooked meatball samples formulated with GG had significantly lower moisture and fat contents than control samples. The moisture content of raw meatball samples decreased when gums (GG, locust bean gum, xanthan gum, and carrageenan) were added.	Meatballs have an increased texture, and finally, panelists preferred GG‐added samples based on sensory analysis.	Demirci et al. (2014)
Sauces and dressings	Tomato ketchup	It is worth mentioning that GG reduced both the serum loss of tomato ketchup and its flow values, thus making it a useful thickener for tomato ketchup.	GG enhanced the consistency of tomato ketchup in a significantly greater way than other hydrocolloids, such as carboxymethyl cellulose, sodium alginate, gum acacia, and pectin.	Gujral et al. (2002)
	Béchamel sauce	The consistency index increased significantly when all hydrocolloids were added to the model system. Applying the GG increased yield stress and the syneresis amount was decreased, which means better quality	GG is useful for making sauces, like Béchamel sauce without forming a gel and for keeping water bound	Heyman et al. (2010)
	Mustard sauce	GG had a strong correlation with the textural properties over the other parameters.	GG is useful for making sauces, like mustard sauce without forming a gel and for keeping water bound	Wang et al. (2016)
	Mayonnaise	The stability of GG–pea protein emulsions had significantly increased. Also, treated samples exhibited excellent emulsifying and rheological properties	Guar gum has almost eight times the thickening power of corn starch	Shen et al. (2022)
	Tomato ketchup	The consistency of GG and xanthan gum‐based tomato ketchup was increased significantly, followed by other hydrocolloids. In terms of serum loss and flow value, xanthan gum and GG caused significant reductions	The perfect consistency can be achieved by adding GG to recipes like tomato ketchup sauce	Shah et al. (2007)
	Soft tofu stew sauce	GG affected the sensorial and rheological properties of soft tofu stew sauce (a Korean sauce) enriched with different levels of GG (0%, 0.1%, 0.2%, 0.4%, and 0.5%). The rheometer monitored the steady flow of stew sauces, and the GG‐added products exhibited Newtonian behaviors at 0.0%, 0.1%, and 0.2%, while 0.4% and 0.5% exhibited pseudoplastic behaviors. Based on rheological testing, they found that 0.1% of GG‐added products were the most popular among consumers	It seems that the addition of GG not only influenced rheological properties but also overall acceptability of the stew sauce	Im et al. (2015)
	Starch paste	Adding GG and xanthan gum to cold‐stored starch pastes results in syneresis reduction	An acceptable functional quality could be achieved by incorporating GG into a starch paste	Mali et al. (2003)
Beverage industry	Carrot juice	Using GG can stabilize the cloudy appearance of carrot juice to keep the carrot juice clear even if it is kept at ambient temperature for up to 6 months	GG can be used to partially replace CMC and improve the stability and physical properties of orange juice	Qin et al. (2005)
	Muskmelon juice	The optimal formulation showed good stability and physical properties, sensory analysis, particle size distribution, and rheological properties	GG stabilized muskmelon juice	Sallaram et al. (2014)
	Fermented milk beverages (FMB)	The quality of GG‐based FMBs preserved and replaced the fat in the formulation	The fermented milk beverages made with guar hydrocolloid were acceptable	Thombare et al. (2016)
	Fermented milk beverages (FMB)	The addition of different hydrocolloids, especially GG, increased firmness, pH, apparent viscosity, protein content, and adhesiveness and reduced syneresis of the FMB	There was a significant influence of the fat content on the texture parameters, and the microstructure of the material was also visualized and supported the findings	Amaral et al. (2018)
Low‐fat foods	Chocolate	As the concentration of GG in the end chocolates increased, the hardness of the end chocolates also increased. The melting behavior of chocolates also followed a similar trend, indicating that melting points increased as cocoa butter levels decreased while the melting points increased simultaneously	GG could be used instead of cocoa butter to create low‐fat chocolate, which would be beneficial for people with diabetes	Amir et al. (2013)
	Low‐fat chicken sausages	Low‐fat chicken sausages that contain GG and xanthan gum would retain acceptable quality even after being stored for 6 months	GG could replace fat in low‐fat meat products	Andres et al. (2006)
	Low‐fat goshtaba	The addition of a 0.5% gum mixture (a 1:1 ratio of GG to xanthan gum) resulted in a low‐fat product with similar sensory and textural properties to those of a high‐fat product when it came to sensory and textural quality	GG in low concentration could replace fat in low‐fat meat products	Rather et al. (2015)
	Yogurt	Using GG to replace the butter resulted in a reduction in syneresis and an improvement in texture, rheology, and rheological characteristics, and it caused low‐fat products to be comparable to those with full fat	GG could replace fat in low‐fat yogurt	Brennan and Tudorica (2008)
	Ice cream	A GG mixture (0.2%) reduced the likelihood of big ice crystals forming in ice cream, and improved body, texture, and the melting properties	GG and basil seeds as a blend can improve the sensory qualities of low‐fat ice cream	Javidi et al. (2016)
	Chocolate	GG‐based chocolate has acceptable organoleptic features, and the final product contained fewer calories when an optimal combination was used	GG could be an effective alternative for fat (cocoa butter) in chocolate	Nazira and Azada (2016)
	Low‐fat frankfurters	Low‐fat frankfurters made with GG were stable in quality compared to other fat substitutes	GG could replace fat in low‐fat meat products	Foegeding and Ramsey (1986)
	Low‐fat carabeef cookies	They found that most physicochemical characteristics changed markedly as GG levels increased. There was an improvement in the sensory scores, including the texture profile, the color profile, and the flavor profile. Despite this, no significant difference was observed in hardness	Most physicochemical characteristics of low‐fat carabeef cookies were significantly changed as GG levels were increased	Goswami et al. (2018)

#### 4.1.1 Bakery goods and pasta

There are several reasons why hydrocolloids, especially GG, are used in bakery products, such as different types of bread, biscuits, cakes, and donuts (Rosell et al., 2001). As guar remains “generally recognized as safe,” GG is used as an additive in numerous foods and beverages in varying amounts as a fiber source. Further, the primary function of this compound is to regulate both the water absorption and the rheology of the dough or batter (Tavakolipour & Kalbasi‐Ashtari, 2007; Venkateswara Rao et al., 1985). It improves mixing and recipe tolerance, extends the shelf life of products by retaining moisture, prevents the formation of syneresis in frozen foods (like frozen bread), and controls spreadability (Ribotta et al., 2004).

Adding GG to wheat bread dough significantly increases the loaf volume once baked (Cawley, 1964). Also, Kohajdová and Karovičová (2008) prepared an experiment to evaluate the effect of different hydrocolloids such as GG, arabic gum, methyl 2‐hydroxyethyl cellulose, and xanthan gum on the final and rheological characteristics of baked goods. By adding these compounds, the water absorption capacity of the dough and stability were increased. Also, sensory acceptance and volume of baked goods were enhanced. While testing bread firmness values during 72 h of storage, baked goods' shelf life was shorter when products containing cellulose derivatives were used. Due to its rheological, sensory, and crumb‐softening properties, GG could be recommended as an improved bread‐making ingredient. For instance, when looking for the reduction of the staling, GG is the best additive due to its softening and retarding the firming of the baked goods crumb effects (Kohajdová & Karovičová, 2008).

In addition to bread, a great deal of popularity is accorded to cakes, donuts, and biscuits among children due to their high nutritional value. Therefore, reducing fat in these products and using suitable alternatives increases their quality and sensory properties (Salehi, 2019). Several reports have documented the use of GG in preparing low‐fat biscuits and cakes (Chugh et al., 2013). It has been reported that rice cakes containing a mixture of xanthan–guar hydrocolloids without an emulsifier and fat were found to be the firmest due to the thickening of the crumb walls near the air spaces in the rice cakes (Turabi et al., 2008). Specifically, GG is used in gluten‐free bakery products to improve nutritional value and crispness (Encina‐Zelada et al., 2019). Gluten in wheat provides elasticity in bakery foods, including bread, which GG substitutes for (Elkhalifa et al., 2007). Moradi et al. (2021) studied the impact of GG addition in gluten‐free (GF) bread formulation from the physicochemical point of view. The multivariate analysis showed that the adhesion, porosity, and elongation of bread samples were increased while the firmness, wrinkle, and specific volume were reduced. Thus, there was an acceptable quality level of the optimal formulation, indicating the potential for industrial use, and to achieve the desired characteristics, 1% GG is recommended for semi‐bulk bread formulations containing potato flour (Moradi et al., 2021).

A low level of GG (2%–5%) can be added to extruded products, such as breakfast cereals, pasta, and snacks, to boost extruder output and prevent retrogradation because of a lubricant effect, improve shape definition, and enhance crispness and crunch. This is achieved by controlling the retrogradation of starch as it is stored during the storage period (Shaikh et al., 2008). Furthermore, GG adds nutrition to cereal bars by stabilizing moisture and binding the dry ingredients, such as fruit and cereal, for an effective texture during storage. In a study by Sandhu et al. (2015), adding GG to pasta dough formulation increased the strength of the dough and improved mechanical and rheological properties (Sandhu et al., 2015).

#### 4.1.2 Dairy products

Because of its versatility, GG can be adapted into various dairy products to fit different purposes such as influencing crystallization, preventing creaming, syneresis or settling, improving the freeze–thaw behavior, and stabilizing foam. GG helps to keep fat ingredients from separating and maintaining homogeneity of texture in preparations such as cheese, yogurt, coconut milk, aerated desserts, toppings, ice cream, and milkshakes by ensuring that thicker ones (such as cream) are uniformly combined with thinner ingredients (such as water) (Lee & Chang, 2016; Mudgil et al., 2017; Saha & Bhattacharya, 2010). It is also important to mention that the main reason for the amazing consistency of your ice cream is that it contains hydrating ingredients such as GG powder and other thickening agents that also add to its texture. GG, in combination with other hydrocolloids like carrageenan, performs well in ice creams that are processed at high temperatures for a limited amount of time due to the short time it takes to hydrate (Mudgil et al., 2014). In this way, it could be helpful in reducing ice cream crystals. For example, adding GG to dairy products like ice cream and yogurt ensures that the products retain their creamy texture, for which the primary purpose is product stabilization. In the study by Julien (1953), guar‐based ice creams with 0.3% levels of GG showed satisfactory results in the cohesion and body of the mix (Julien, 1953). Additionally, a GG mixture reduces the likelihood of big ice crystals forming in ice cream and other frozen desserts. However, due to their unique structure, which is associated with their viscosity, xanthan gum, and GG cannot be mixed because xanthan gum is a low‐viscosity gum often used in applications with lower viscosity, such as in salad dressings and sauces (Demirci et al., 2014).

Guar gum is similarly added to yogurt to stabilize it and is also used to prepare products containing high levels of dietary fiber as well as low‐fat yogurt. According to Lee and Chang (2016), adding GG to yogurt dramatically improved the rheological characteristics of stirred yogurt (Lee & Chang, 2016). Brennan and Tudorica (2008) demonstrated successfully that GG could replace fat in low‐fat yogurt. Using GG to replace the butter resulted in a reduction in syneresis and an improvement in texture, rheology, and rheological characteristics, resulting in low‐fat products comparable to those with full fat. On the other hand, there is an acceptable level of sensory acceptance for low‐fat products containing GG as a fat substitute. Mudgil et al. (2016) revealed that an acceptable functional and sensory quality could be achieved by incorporating PHGG into yogurt to enrich the soluble fiber content, and according to the study conducted by Rezaei et al. (2011) on the effect of the addition of the combination of GG and arabic gum in yogurt formula, the GG (0.2%)–arabic gum (0.5%) mixture presented the highest acceptability score and the highest total acceptability score (Rezaei et al., 2011).

Furthermore, GG contributes to improving the texture and body of cheese by controlling syneresis through water‐phase management, which regulates weeping from the cheese (Gupta & Variyar, 2018). Hesarinejad et al. (2021) evaluated the impacts of temperature and two different selected hydrocolloids (GG and gelatin) on the appearance, rheological, physicochemical, and textural features of ricotta cheese. Due to the interconnected gel‐like structures of the ricotta cheese and the addition of 5% gelatin and 1% GG, the viscoelastic characteristics of the cheese exhibited a shear‐thinning (pseudoplastic) behavior under shear loading. Also, the highest elastic modulus was achieved at a combination of 5% and 0.5% of gelatin and GG, respectively. Finally, mixing gelatin with GG was a better choice to improve the gel strength (Hesarinejad et al., 2021).

Guar gum is also used to provide a smooth texture to products such as doogh (Im et al., 2015). Considering the viscosity of the doogh, Pirsa et al. (2018) found that the best conditions were achieved on the first day of the doogh production with 0.1% (w/w) of quince seed mucilage (Qm) and 0.2% (w/w) of GG. The results showed significant differences between the sensory and physicochemical properties of the newly formulated doogh due to each factor (storage time, GG%, and Qm%) (Pirsa et al., 2018).

#### 4.1.3 Meat products

Guar gum is widely used in the meat industry as a fat replacement as well as in edible films to improve shelf life and extend the shelf life of meat products. A study by Foegeding and Ramsey (1986) demonstrated that low‐fat frankfurters made with GG were stable in quality compared to other fat substitutes (Foegeding & Ramsey, 1986). Andres et al. (2006) investigated how xanthan and GG can be used as hydrocolloids in preparing low‐fat chicken sausages, and it was demonstrated that these products would retain acceptable quality even after being stored for 6 months (Andres et al., 2006). Another experiment was conducted on preparing low‐fat goshtaba (a traditional Indian meat product), and it was determined that adding a 0.5% gum mixture (a 1:1 ratio of GG to xanthan gum) resulted in a low‐fat product with similar sensory and textural properties to those of a high‐fat product when it came to sensory and textural quality (Rather et al., 2015).

Guar gum absorbs water in the gut, producing a mild laxative effect (Feiner, 2006). As a result of its performance in water binding, GG is soluble in cold and hot water, making it easy to be absorbed. As a thickening agent in processed meat products, it primarily controls syneresis in the products, prevents fat migration during storage, and regulates viscosity and rheology (Mudgil et al., 2014). An algin/calcium and a salt/phosphate structure beef roll containing GG have been shown to exhibit an improved water‐holding capacity (Shand et al., 1993). Further, hydrocolloid gums (GG) store water and create a gel network, which enhances their juiciness over time (Gupta & Variyar, 2018). It was investigated whether GG and carrageenan could replace phosphate in processing pork sausages. In addition to increasing water‐holding capacity, the hydrocolloid compounds also reduced cooking loss. Compared to the control sausages, hardness, cohesiveness, and chewiness were well maintained. Furthermore, phosphate‐free sausages were stable for extended periods (Park et al., 2008).

Regarding the importance of textural characteristics of meat products, the effects that can be obtained from supplementing low‐fat, high‐moisture meat batters with kappa‐carrageenan, GG, xanthan, locust bean gum, methylcellulose, and a kappa‐carrageenan/locust bean gum combination have been assessed by Foegeding and Ramsey (1986). The texture profile analysis revealed changes in the texture properties of GG‐based batter samples, which was better than controls. It was also found that low‐fat frankfurters were just as acceptable as control frankfurters after sensory evaluation (Foegeding & Ramsey, 1986). Goswami and his co‐workers demonstrated that most physicochemical characteristics of low‐fat carabeef cookies were significantly changed as GG levels were increased. They found that most physicochemical characteristics changed markedly as GG levels increased. There was an improvement in the sensory scores, including the texture profile, the color profile, and the flavor profile. Despite this, no significant difference was observed in hardness (Goswami et al., 2018).

#### 4.1.4 Sauces and dressings

In salad dressing, it can serve as a thickener at about 0.2%–0.8% of the total weight of the dressing due to its high dispersibility in cold water and compatibility with acidic emulsions (Mudgil et al., 2014). Increasing the viscosity of the water phase in salad dressings decreases separation rates between oil and water (JFM, 1974). Much research has been conducted on using GG as a thickener for pickle and relishes sauces as an alternative to tragacanth (Burrell, 1958). The reason for using GG is that other hydrocolloids cannot impart these properties to salad dressings. In contrast to Konjac glucomannan, GG dissolves more easily in cold water than in hot water. This can make it more difficult to use Konjac glucomannan in certain food applications, such as cold beverages or salad dressings (Ran et al., 2022). A study by the American Chemical Society found that GG enhanced the consistency of tomato ketchup in a significantly greater way than other hydrocolloids, such as carboxymethyl cellulose, sodium alginate, gum acacia, and pectin. It is worth mentioning that GG reduces both the serum loss of tomato ketchup and its flow values, thus making it a useful thickener for tomato ketchup (Gujral et al., 2002). Moreover, the stability and other physicochemical properties of béchamel sauce were evaluated in different concentrations of GG, carboxymethylcellulose, and xanthan gum. The consistency index increased significantly when all hydrocolloids were added to the model system. Applying the GG increased yield stress, and the syneresis amount was decreased, which means better quality (Heyman et al., 2010).

Several studies have been published on the textural and rheological characteristics of GG‐based sauces. It is known that rheological properties are a measure of food quality, which reflect the flow and deformation patterns as well as the behavior of substances at the interface between solids and fluids (Burrell, 1958). In this regard, the rheological measurements of mustard sauce samples showed weak gel‐like rheology and a strong shear thinning behavior when subjected to shear forces. Several parameters were evaluated to investigate the textural properties of the different materials, including springiness, hardness, gumminess, spreadability, and cohesiveness. The results showed that GG strongly correlated with the textural properties over the other parameters (Wang et al., 2016). Shen et al. (2022) supplemented mayonnaise samples with pea protein and GG (G‐PPI), and then they found that the stability of G‐PPI emulsions had significantly increased. Also, treated samples exhibited excellent emulsifying and rheological properties (Shen et al., 2022).

To control the viscosity of ketchup, hydrocolloid thickeners are often used to control its shear‐thinning flow and tendency to yield. Shah et al. (2007) illustrated that the consistency of GG and xanthan gum‐based tomato ketchup was increased significantly, followed by other hydrocolloids. In terms of serum loss and flow value, xanthan gum and GG caused the greatest reductions (Shah et al., 2007). Similarly, Sahin and Ozdemir (2004) pointed out that the consistency of GG‐based formulated ketchup samples was increased more than others (tragacanth, carboxymethyl cellulose, and xanthan gum) (Sahin & Ozdemir, 2004).

#### 4.1.5 Beverage industry

There are many applications of gums in the beverage industry, including thickener and viscosity enhancers (Gupta & Variyar, 2018), but when it comes to safety, GG is the best selection. However, some studies have suggested that carrageenan may cause inflammation and digestive issues in animals and humans, although the evidence is not conclusive. However, it is important to note that carrageenan has been deemed safe for consumption by regulatory agencies such as the FDA and the European Food Safety Authority (EFSA) (Ulu, 2006). Also, GG is easier to extract and produce than carrageenan, which requires a more complicated extraction process (Sow et al., 2018). Combination of GG and xanthan gum synergistically affect solution viscosity in food systems (Quinzio et al., 2018). The result of its stability at a pH level lower than that generally found in beverages, as well as its solubility in cold water, makes it a great choice for the beverage industry. This is also because it is a tasteless and odorless molecule, so it adds no taste or flavor to the beverage it is added to, and it does not make the beverage taste or smell any worse (Chudzikowski, 1971). Typically, GG is added to diet drinks to improve the mouthfeel and body of the beverage in a range between 0.10% and 0.12% (w/v) level, depending on the formulation. In addition, the amount of GG required to stabilize pulp in juices ranges from 0.25 to 0.75 (w/v). Using GG can stabilize the cloudy appearance of carrot juice to keep the carrot juice clear even if it is kept at ambient temperature for up to 6 months (Qin et al., 2005). Additionally, GG was used to stabilize musk melon juice (Sallaram et al., 2014).

During the shelf life of fermented milk beverages (FMBs), hydrocolloids, for example, gum arabic and GG, can be added to preserve the quality and replace the fat in the formulation (Thombare et al., 2016). In this way, Amaral et al. (2018) found that the addition of different hydrocolloids, especially GG, increased firmness, pH, apparent viscosity, protein content, and adhesiveness and reduced syneresis of the FMB. There was a significant influence of the fat content on the texture parameters, and the microstructure of the material was also visualized and supported the findings (Amaral et al., 2018). In addition to serving as a soluble fiber source, GG is added to beverages for a healthy digestive system (Lyly et al., 2009). Healthy, non‐diabetic volunteers have been shown to have a significant reduction in peak glucose levels following the consumption of beverages containing GG (Wolf et al., 2003).

#### 4.1.6 Low‐fat foods

A primary goal of the food industry is the development of new types of products, which is accomplished by incorporating various ingredients into formulations to achieve this goal. For example, some are reduced‐fat/sugar products (such as reduced‐fat spreads, biscuits, and sugar‐free beverages) or enriched foods (like enriched cakes or milk with vitamins and minerals). Among the mentioned cases, spreads, which include a wide range of daily foods, such as dairy products (low‐fat cheese or low‐fat yogurt), play a special role. A gel composed of lipid droplets emulsified into a gel matrix is called a spread (Lee & Klostermeyer, 2001; Mcclements & Demetriades, 1998). Spreads can be called emulsion‐filled gels, emulgels, or emulsion hydrogels because the gel matrix is encapsulated with emulsified lipid droplets (Lu et al., 2019).

It is common to find emulsion gels in food products such as spreadable chocolates (Nazira & Azada, 2016), cheese (Swenson et al., 2000), desserts (Jooyandeh et al., 2019), butter (Panchal & Bhandari, 2020), and even meat products (sausages) (Kumar, 2021) that have been reformulated (Lamprecht et al., 2001). It has been shown that emulsion gels are suitable for developing fat‐reduced foods and offer a unique option (Chung et al., 2016) since they can be adjusted in terms of textural properties and have a reduced lipolysis rate (Dickinson, 2012; Guo et al., 2017; Mao et al., 2018). However, they are thermodynamically unstable due to a large interfacial area between water (dispersed phase) and oil (continuous phase). Using specific ingredients such as thickening agents (e.g., surfactants, proteins, and polymers (hydrocolloids)) can result in kinetically stable emulsions (Yong et al., 2021). Several studies have found that GG stabilizes emulsions by increasing the concentration of droplets and the viscosity, leading to more closely packed droplets (Dickinson, 2012). On the other hand, the apparent viscosity of liquids is associated with the inherent viscosity power of guar products, ranging from 0.7 to 15.0 D/g. This is why GG is often used in systems where fat levels are reduced or eliminated by replacing them with fat. In addition, GG dissolves readily in cold water, whereas other hydrocolloids like alginate (Sow, Toh, et al., 2019) and gellan (Sow, Tan, & Yang, 2019) require warm or hot water to fully dissolve. This means that GG can be added directly to cold liquids to thicken them, while alginate requires some level of heat to fully incorporate into a recipe. GG is also more resistant to acidic conditions than alginate, which can break down in acidic environments (Mcclements & Demetriades, 1998).

It was discovered that a xanthan gum/GG hybrid blend could substitute cocoa butter in the developed low‐fat chocolate. As the concentration of hybrid polysaccharides in the end chocolates increased, the hardness of the end chocolates also increased. The melting behavior of chocolates also followed a similar trend, indicating that melting points increased as cocoa butter levels decreased while the melting points increased simultaneously. It was concluded, however, that polysaccharides could be used instead of cocoa butter to create low‐fat chocolate, which would be beneficial for people with diabetes (Amir et al., 2013). Nazira & Azada, 2016 also concluded in the same result that GG‐based chocolate has acceptable organoleptic features, and the final product contained fewer calories when an optimal combination was used.

### 4.2 Novel applications

#### 4.2.1 Films and coatings

Researchers are currently trying to find a safe way to preserve food commodities without damaging the environment and as safe as possible for human consumption (Naeem et al., 2019). Even with the use of modern technology, post‐harvest losses occur frequently. A cost‐effective approach to enhancing fruit shelf life is by applying films and coatings (Cardello, 2003). The food supply chain has become increasingly dependent on food packaging. As a result, packaging becomes an essential step once foods have been minimally processed or fully processed. It allows them to be transported from the factory to the market or retailer and provides the needed information about the product while allowing product commercialization and distribution (Díaz‐Montes & Castro‐Muñoz, 2021). Such materials are able to enhance the food's physicochemical, functional, and organoleptic properties (Kumar et al., 2021). A variety of packaging materials have been employed, including paper, cardboard, metal, glass, and plastic. However, urban solid waste (USW) is likely to be the result of this traditional preservation method, and there have been limitations to conventional food packaging materials like plastic (such as polyethylene, polypropylene, and polyethylene terephthalate), paper, and some metals (aluminum) (Han, 2005; Kester & Fennema, 1986). Researchers and manufacturers are forced to adopt bio‐based sustainable packaging materials and circular economic processes due to consumers' changing attitudes toward food safety and quality (Baldwin et al., 1996; Bourtoom, 2008).

**FIGURE 4:**
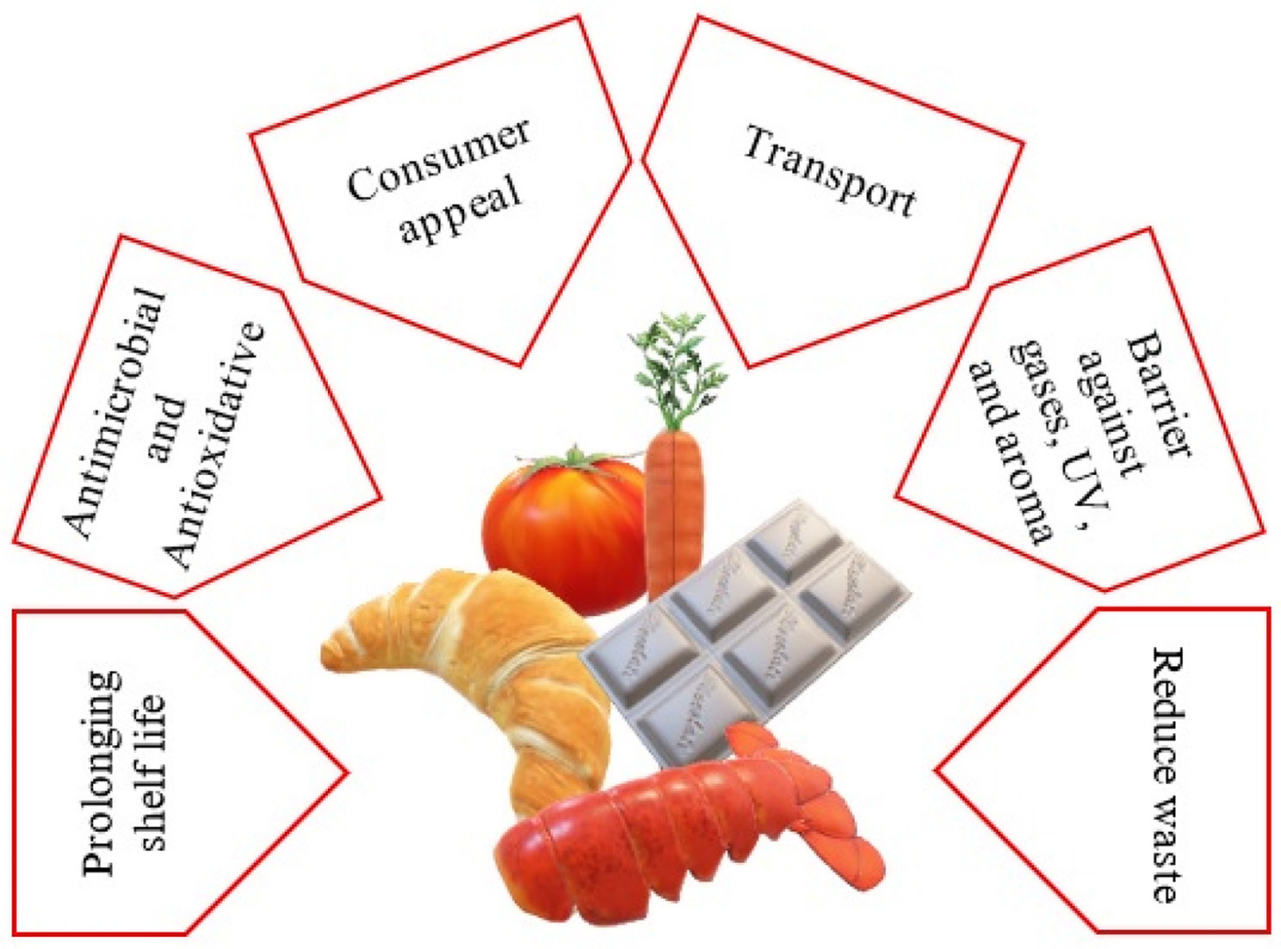
Scheme illustrating the main properties of films and coatings.

Future eco‐friendly food packaging alternatives could include biodegradable packaging. It is possible to increase the shelf life of food and reduce waste to a certain extent using biodegradable packaging (Krochta, 2017). In addition to extending post‐harvest life, these coatings are a cost‐effective way to keep production costs low; however, they have several limitations because of their poor mechanical properties (Nieto, 2009). Due to this, biodegradable packaging, especially edible coatings, has been studied extensively in recent years to minimize pollution problems (Kester & Fennema, 1986; Raghav et al., 2016). The term edible film or coating refers to any material less than 0.3 mm thick, composed of biopolymers dispersed in an aqueous solution (Han, 2005). It is relatively easy to produce, recycle, and degrade. It is generally accepted that most edible packaging substances are made of natural polymeric materials that aim to preserve the quality and prolong the life of minimally processed products, such as fruits and vegetables, in a short time (Stoica et al., 2013). Figure 4 describes the main aspects that edible films and coatings can present (Salehi, 2020).

A wide variety of edible packaging materials can be used for the edible packaging of beverages, vegetables, fruits, and meat items; however, it is necessary to dissolve film materials in solvents such as water, alcohol, or a mixture of water and alcohol during the manufacturing process (Díaz‐Montes & Castro‐Muñoz, 2021). Three groups of materials can be distinguished: hydrocolloids (including gums and proteins), lipids, and composites (Han, 2005). Due to their physicochemical and rheological features, polysaccharides (chitosan, GG, xanthan gum, etc.) have become increasingly popular in the biodegradable food packaging industry. In this regard, gums might be a good choice for improving the sustainability of biodegradable packaging due to their polymeric structure and accessibility (Thombare et al., 2016). In addition to soluble solutes, drugs, flavorings, and other active ingredients, gum polymers can hold insoluble particulates. Aside from their biological origin, gum molecules display significant differences in their linear chain lengths, molecular weights, and branching properties (Liu et al., 2020). They also have good mechanical strength, which makes them acceptable barrier materials to atmospheric gases. For controlling water vapor transfer through a food packaging film, water vapor permeability (WVP) is a critical performance property (Alizadeh‐Sani et al., 2020). Furthermore, gums can be combined with other polymers, such as proteins and lipids, to alter a film's solubility and permeability to water vapor and oxygen (Salehi, 2020). According to a general rule of thumb, polysaccharide gums with a high molecular weight and linear structure form thick films. In this way, there are different gums, but linear and neutral polysaccharides have the best film‐forming properties and provide the strongest films (Lacroix & Vu, 2014).

Gums are complex polysaccharides that consist of monomers or polymers of various molecular weights and conformations linked together (Cerqueira et al., 2017). These polymers belong to the family of hydrocolloids, such as xanthan, guar, and konjac, which have a high amount of free OH groups (Kester & Fennema, 1986). Gum polymers come in various combinations that can be used in filmmaking (Salehi, 2020). In addition to konjac, guar, Arabic, xanthan, curdlan, and pullulan, many gum polymers have molecular weights (MWs) over 1 million; yet, their film‐forming property varies as much as their thickening property. Several factors that affect their film‐forming properties include structural conformation, molecular weight, the ionic charge on the molecule, and steric groups. Among many polysaccharides that can form films, GG is a good example, whereas polymers with highly branched chains (e.g., gum Arabic, gum ghatti, and larch gum), either with or without an anionic charge, form weak films that peel or flake off when removed from casting surfaces (Gupta & Variyar, 2018; Madni et al., 2021).

In recent years, GG has also been suggested for the preparation of edible coatings and dips due to its flocculation, thickening, emulsification, and film‐forming capabilities (Rahman et al., 2021). It is because the GG contains galactomannan, which is composed of straight chains of galactose and mannose, that alternately split into long chains. GG conveys galactose‐to‐mannose components in percentages ranging from 1:1 to 1.8:1 (Ruelas‐Chacon et al., 2017). The strong hydrogen bonding properties of this compound are attributed to its easy solubility in cold and hot water. Aside from its high water solubility in a wide range of protective colloids, GG is also non‐toxic, available, low in cost, biodegradable, and occurs in solvent‐resistant films (Thombare et al., 2016). All these factors are responsible for their diverse functions when used in pharma, foods, and other industries (Liu et al., 2020). In addition to possessing antibacterial properties, GG can improve the antimicrobial characteristics of films. In this way, Arfat et al. (2017) explored the effects of GG–silver–copper nanocomposites against food‐borne pathogenic bacteria strains. As a result of the fabrication of the membrane, it showed excellent antimicrobial potential against *Listeria monocytogenes* as well as *Salmonella enterica* sv. *Typhimurium* (Arfat et al., 2017). It has been demonstrated through a study (Kundu et al., 2017) that the addition of GG to gelatin film significantly improves its transparency due to the interaction between polysaccharides and proteins. Furthermore, it has also enhanced the thermal insulation properties of the membrane as well as its antimicrobial properties. Because of these critical characteristics, it has been suggested that the membrane can be used as edible food packaging. This section aims to evaluate the various capabilities of GG‐based coatings in different industries (Table 2).

**TABLE 2:** Various guar gum‐based coatings, diverse applications, and results.

Film	Properties	Applications	Results	References
GG‐based edible coatings supplemented with spice extracts	According to the results, a significant difference was not found between the treated and control lemons regarding physicochemical attributes.	For lemons during cold storage (10°C)	Compared to all other treatments, fennel‐coated lemon extracts had an increased shelf life of 24 weeks at 10°C. Treatment also reduced the bacterial load of lemons as well. In conclusion, cold‐storing lemons could be improved by coatings containing GG fortified with extracts	Naeem et al. (2019)
SC/GG/TiO_2_/cumin essential oil nanocomposite film	They found that the addition of GG and TiO_2_ significantly enhanced the mechanical (such as flexibility and tensile strength (TS)) and the water vapor permeability (WVP) of the composite. Water vapor diffusion followed by WVP of SC/GG composite film is decreased due to blending SC with GG. On the other hand, SC/GG/TiO_2_ films exhibited notable antibacterial activity against *E. coli* O157: H7, *S. aureus*, and *S. enteritidis*.	For packaging application	Films incorporating SC/GG, TiO_2_, and CEO are suitable for food packaging and wound dressing, among other applications.	Alizadeh‐Sani et al. (2020)
CH/GG‐based composite film	—	For shiitake mushrooms during cold storage	Compared to the mushrooms without any coating (control), mushrooms coated with CH (1%) + GG (15%) maintained higher tissue firmness and reduced electrolyte leakage. Similarly, sensory evaluation was used to identify whether CH (1%) + GG (15%) coating improved the shiitake mushroom's overall quality.	Huang et al. (2019)
GG/CMC incorporated with LSE and HNT	Analysis showed that the flexibility and elongation at break (E) of GG/CMC incorporated with 20% LSE were significantly enhanced (from 29.93% to 62.12%); however, TS was reduced with the addition of LSE. On the other hand, LSE significantly improved the UV–light resistance of GG/carboxymethyl cellulose films. In addition, the GG/CMC/LSE film illustrated high antioxidative activity, approximately a 10 times increase compared to GG/CMC films (from 9.46% to 91.52%).	For roasted peanuts during storage	Improved safety of roasted peanuts over 8 days and the packaging film could be used potentially for packaging oxygen‐sensitive foods with low water activity to preserve and extend their shelf life because of the excellent compatibility and porous surface of GG/CMC/LSE 10% and 20% films compared to control films (without LSE).	Kshirsagar et al. (2022)
GG/potassium sorbate	—	For apples and cucumbers	This formulation delayed the fungal activity and extended the shelf life of fruits.	Mehyar et al. (2014)
CS/GG/PVA cross‐linked HCA	The mechanical properties of active composite improved, and WVP was decreased due to the strong compatibility among CS/GG/PVA/HCA film components. Also, it showed potential antimicrobial features against *E. coli* and *S. aureus* bacteria due to the presence of GG and HCA.	For packaging application	It could be chosen as an alternative to plastic packaging in the food packaging industry.	Bhat et al. (2021)
Modified GG (GGBA)	In addition to being resistant to water, the new GGBA also gave good results in organic solvents such as dimethyl sulfoxide, which are not aqueous. As a result, it had a low WVP with a high water contact angle (90.35°) which is a good choice for hydrosensitive foods. Furthermore, when applied in a stand‐alone condition or as a coating, the mechanical properties of the film represent the effectiveness and reliability of the coating. Finally, inhibition was observed against both Gram‐positive and Gram‐negative organisms, suggesting that hydrophobic contact is responsible.	For packaging application	It could be chosen as an alternative to plastic packaging in the food packaging industry.	Das, De, et al. (2021)
GGIA‐keratin film	As a result of the protein–polysaccharide association, the mechanical properties of the film were improved as well as physicochemical properties. It showed high compatibility and TS for human dermal fibroblast cultivation. Moreover, the standardized film demonstrated superior antimicrobial activity against Gram‐positive bacteria as well as Gram‐negative bacteria compared with commercially available films.	For skin tissue engineering applications	It showed good physicochemical and antimicrobial potential for human dermal fibroblast cultivation.	Das, Das, et al. (2021)
GG/polyhydroxyalkanoates/curcumin	There was an improved rigidity of the composite that was prepared due to the hydrogen bonds formed between GG and polyhydroxyalkanoates. A further finding of the study was that the roughness of the surface of the film was a positive factor in favoring cellular attachment and proliferation, which helped accelerate the healing process of the wound.	For wound healing	Excellent rigidity and roughness of the surface of the film were helpful for wound healing.	Manna et al. (2015)
Agarose/GG/polyaniline composite film	The obtained film displayed enhanced mechanical and electrical properties, suggesting that it would suit electrochemical instruments and sensor designs.	For use in electrochemical instruments and sensor designs	The film displayed enhanced mechanical and electrical properties for electrochemical uses.	Vaghela et al. (2014)
Robust GG/cellulose nanofibrils film	The membrane was both strong and had excellent oxygen barrier properties.	For packaging application	It could be chosen as an alternative to plastic packaging in the food packaging industry.	Dai et al. (2017)
Cross‐linked GG/chitosan composite film	In addition to good mechanical strength, the film illustrated low WVP and high water contact angle (92.8°) (hydrophobic), showing potential stability against water and water vapor compared to the chitosan film.	For packaging application	It could be chosen as an alternative to plastic packaging in the food packaging industry.	Rahman et al. (2021)
PVA/GG/Ag‐based nanocomposite film	It showed an excellent TS (23.93 MPa) compared to PVA film. Ag NPs enhanced the hydrophobicity properties, caused low permeability in water vapor and oxygen, and resulted in good antimicrobial activity against *E. coli* and *S. aureus* bacteria. It was noteworthy that it did not observe any migration from the nanocomposite film through food matric.	For food packaging	It could be chosen as an alternative to plastic packaging in the food packaging industry.	Gasti et al. (2021)
Pea starch/GG edible film	According to the solubility, transparency, and moisture content results, the optimum formulation was 2.5 g pea starch, 0.3 g GG, and 25% glycerol, demonstrating excellent packaging properties for different foods. However, due to the lack of data on the mechanism of action of this edible film in preserving food, it remains unclear and needs to be investigated.	For food packaging	It could be chosen as an alternative to plastic packaging in the food packaging industry.	Saberi et al. (2017)
CMC/GG/Ag NPs bio‐nanocomposite film	—	For packaging of strawberries	Antimicrobial‐packaged strawberries lost much less weight than control‐packaged strawberries. Compared to commercial plastic films and control films incorporating Ag NPs, the film incorporating GG and Ag NPs had a better preservation effect and enhanced the shelf life of fresh strawberries.	He et al. (2021)

In addition to improving their shelf life, edible films and coatings provide sensory and nutritional benefits to food products (Galus et al., 2020). As a result, Huang et al. (2019) evaluated the effect of chitosan (CH)/GG‐based composite film on shiitake mushroom quality during storage at 4 ± 1°C for 16 days. To dissolve the equal amounts of CH (1%, w/v) and GG (1%, w/v) solutions, the solutions were stirred at room temperature for 1 hour with 1% acetic acid, and 0.75 glycerol plasticizer was added. To prepare film samples, they used the casting method. Compared to the mushrooms without any coating (control), mushrooms coated with CH (1%) + GG (15%) maintained higher tissue firmness and reduced electrolyte leakage. Similarly, sensory evaluation was used to identify whether CH (1%) + GG (15%) coating improved the shiitake mushroom's overall quality. Therefore, CH 1% + GG 15% coatings could be used commercially as a novel preservation method for maintaining shiitake mushroom quality over a long period at 4°C (Huang et al., 2019).

As mentioned before, GG has antimicrobial properties, and using edible films and coatings that contain antimicrobial agents potentially prolongs the shelf life of the food and modifies the film features (Kumar et al., 2021). In this way, Mehyar et al. (2014) developed edible antimicrobial films based on GG and potassium sorbate for fresh fruits (apples and cucumbers), with effective antifungal and antimicrobial activity of both GG and potassium sorbate through microorganisms spoilage (Mehyar et al., 2014). Bhat et al. (2021) fabricated chitosan (CS)/GG/polyvinyl alcohol (PVA) cross‐linked with hydroxy citric acid (HCA) by solution casting method. In the presence of a certain level of GG (0.2%), the effect of HCA has been investigated on different CS/PVA ratios (1:3, 1:1, and 3:1). They found that mechanical properties of active composite improved, and WVP was decreased due to the strong compatibility among CS/GG/PVA/HCA film components. Also, it showed potential antimicrobial features against *E. coli* and *S. aureus* bacteria due to the presence of GG and HCA (Bhat et al., 2021).

Besides food packaging, there are other important uses for guar‐based films. For example, films are used for wound healing and skin repair (Das, Das, et al., 2021; Galus et al., 2020). For instance, GG ester/chicken feather keratin interact film was developed for skin tissue engineering applications. Das, Das, et al. (2021) synthesized guar gum indole acetate (GGIA) biopolymer and cross‐linked it with hydrolyzed keratin from chicken feather waste. As a result of the protein–polysaccharide association, the mechanical properties of the film were improved as well as physicochemical properties. It showed high compatibility and TS for human dermal fibroblast cultivation. Moreover, the standardized film demonstrated superior antimicrobial activity against Gram‐positive bacteria as well as Gram‐negative bacteria compared with commercially available films. Generally, the proposed GGIA–keratin film showed promise as a tool for skin tissue engineering (Das, Das, et al., 2021).

In addition to reducing weight loss, bio‐nanocomposite films/coatings act as an effective barrier against moisture loss (George, 2012). Edible films and coatings can contain a blend of polysaccharides, proteins, and/or lipids called heterogeneous (composite) and can also be used for multilayer packaging. A composite/nanocomposite (NC) is composed of several phases, one of which has a nanoscale dimension (<100 nm) or a material that contains repeat distances of nanoscale among its diverse fragments (Shapi'i & Othman, 2016). Various NC materials have been proposed in the study by Beyene and Ambaye (2019). Some include carbon/ceramic NCs, carbon/polymer NCs, metal/ceramic NCs, metal/metal NCs, ceramic/ceramic NCs, polymer/ceramic NCs, and non‐polymeric‐based NCs. Such films are produced to improve permeability or mechanical properties. In this way, different classes of film formers can be utilized according to their functional characteristics (Kester & Fennema, 1986). Its remarkable blend of properties makes GG NCs derivatives extremely popular in several industries. Throughout their assembly for industrial purposes, they proved to be of valuable assistance (Davachi & Shekarabi, 2018). Gasti et al. (2021), for example, prepared PVA/GG/Ag‐based nanocomposite film for food packaging. It showed an excellent TS compared to PVA film. Ag NPs enhanced the hydrophobicity properties, caused low water vapor and oxygen permeability oxygen, and resulted in good antimicrobial activity against *E. coli* and *S. aureus* bacteria. Notably, it did not observe any migration from the nanocomposite film through food matric (Gasti et al., 2021).

#### 4.2.2 Delivery system

Food products with functional ingredients are becoming more popular as people become more concerned with their health. The water solubility, chemical stability, and adsorption properties of many lipophilic ingredients (unsaturated fatty acids, vitamins, and carotenoids) make them difficult to incorporate into food directly (Fathi et al., 2012). This is why scientists are attempting to find a solution to the challenges they are facing. The delivery of functional ingredients (encapsulation, emulsion gels, powder particles, etc.) can be enhanced during digestion by modifying their dispersibility status in the food matrix, controlling their release, and enhancing their bioavailability (Mcclements & Li, 2010; Rafiee et al., 2019; Velikov, 2012).

##### Emulsion gel

In recent years, emulsion gels have emerged as an interesting delivery method (Dickinson, 2012; Lu et al., 2019; Torres et al., 2016). Various food products, such as emulsion gels, are regularly used in the production process because they possess the properties of both emulsions and biopolymer gels (Guo et al., 2017). Since the beginning of the decade, many studies have demonstrated that emulsion gels effectively deliver minerals, flavors, phenolic compounds, vitamins, unsaturated fatty acids, and other functional elements (Lu et al., 2019). Functional ingredients, for example, are usually more stable when distributed in gels since the gels protect them, and the gel network immobilizes them (Mao et al., 2017). This is why functional ingredients incorporated in the formulation of emulsion gels have a good level of stability (Matalanis et al., 2011).

Proteins [119], polysaccharides, or synergistic mixtures are usually used to prepare emulsion gels (Lopes et al., 2017). The properties and functions of the systems are dependent on the characteristics of the oil droplets. Using a controlled physical structure of emulsion gels, including their digestion behavior in the digestive tract, it is possible to manufacture them to behave differently in different environments (Mcclements & Li, 2010). By manipulating their stability and release behaviors, functional ingredients can be enhanced in their bioavailability by adjusting their stability and release behaviors (Freire et al., 2017). Moreover, by varying the oil/water phase fractions during the gel formation process and the droplet distribution, gels can be obtained that both possess the desired textural and rheological properties and allow controlled delivery of the incorporated ingredients (Mao et al., 2017). One of the critical features of emulsion gels is their stability and availability. These features can be achieved by the addition of particular food additives (such as hydrocolloids, e.g., GG and xanthan gum) adjusting droplet size and viscosity (Lu et al., 2019; Mcclements & Li, 2010).

There are several novel structure emulsion systems developed in response to this need. One of them is multiple emulsions (Esfanjani et al., 2017), another is multilayer emulsions (Huang et al., 2018), a third is solid lipid particles (Wang et al., 2018), and a fourth is nano‐emulsions (Kwan & Davidov‐Pardo, 2018). There have been many reviews of emulsions as a carrier for functional ingredients. Mao et al. explored the relationship between the rheological properties and flavor release of emulsion gels, and they found that flavor released at lower rates and had lower air–gel partition coefficients in emulsion‐filled protein gels than those in ungelled counterparts. In addition, they showed that three fat substitutes, including GG, MCT, and maltodextrin, could enhance the elasticity of the systems. At the same time, the magnitude was mainly affected by the nature and content of the substitutes. With the aid of the fat substitutes, the emulsion gels with lower oil content could have lower air–gel partition coefficients of the flavor compounds, particularly the more lipophilic ones (de Souza Paglarini et al., 2018; Mao et al., 2014). Also, they found that partly replacing the oil phase of an emulsion gel with medium‐chain triglyceride, guar gum, or maltodextrin could enhance the gel strength and slow the release of tested volatile compounds (Mao et al., 2018).

Probiotics and drugs can be delivered using natural gum‐modified emulsion gel. Pandey et al. (2016) found that emulsion gels were suitable carriers for the co‐delivery of probiotics, and their higher viability could be obtained by encapsulating the cells in emulsion gels containing GG and xanthan gum (Pandey et al., 2016). They revealed the combination of natural gum‐modified sunflower oil (41%)‐based emulsion gels for the delivery of probiotics like *Lactobacillus Plantarum* 299v at different storage conditions (4°C, −20°C, and −196°C) was found suitable. It was also recognized that gelled emulsions could be used for the delivery of essential oils (like rosemary essential oil) (Nourbehesht et al., 2018), capsaicinoids [60], anthocyanins [12], lactoferrin [20], terbinafine HCl [47], and flaxseed oil [49], which are shown in Table 3.

**TABLE 3:** Widespread uses of guar gum as an encapsulation agent for food ingredients.

Encapsulated substance	Wall materials	Results	References
Curcumin	GG and cationic guar gum (CGG)	It was found that CGG was more effective at stabilizing structures than GG by decreasing membrane fluidity and enhancing the lateral packing of lipids. Also, it could effectively protect curcumin from the damaging effects of oxidation or heat.	Pu et al. (2019)
Anthocyanin from chokeberry fruit	GG, arabic gum, inulin, and β‐glucan	GG‐based wall covering microencapsulation is useful to preserve the stability of anthocyanins with limited stability.	Pieczykolan and Kurek (2019)
Carotenoid (β‐carotene)	Xanthan gum and GG	The gum mixture proved highly effective in preventing the aggregation of β‐carotene‐loaded liposomes during storage, indicating that it is possible to develop a functional yogurt with β‐carotene encapsulated in liposomes.	Toniazzo et al. (2014)
Fish liver oil	Maltodextrin, sunflower protein, and GG	GG‐covered capsules showed high sensorial quality and antioxidative stability as a capsule cover.	Ogrodowska et al. (2020)
Probiotic bacteria (*L. rhamnoses*)	Whey protein, alginate, and GG	Probiotic‐loaded microcapsules with alginate and also GG covering had the potential to generate several health benefits, and they were more stable during 32 days of storage.	Reid et al. (2007)
Spirulina (as a food supplement)	Alginate and GG	Findings showed that the antioxidative activity of Spirulina was preserved during adverse pHs (like 1.2) and the microencapsulation matrix prevented denaturation at high temperatures (100°C).	Guarienti et al. (2021)
Grape skin phenolic extract	Gum arabic and PHGG	Microcapsules had better physicochemical properties compared to free extract.	Kuck and Noreña (2016)
Quercetin	GG	GG was able to preserve the active compound of quercetin during its passage through the upper part of the gastrointestinal tract (GI) after it was encapsulated.	Singhal et al. (2011)
Mint oil	GG	GG‐based encapsulation of mint oil using spray drying improved the quality and bioactivity during storage time.	Sarkar et al. (2013)
Cardamom flavor (including D‐limonene, 1,8 cineole, myrcene, terpineol, and linalool)	Whey protein isolate (WPI), GG, and carrageenan	There was better retention of 1,8‐cineole and D‐limonene during storage when 30% WPI and 30% WPI + GG were used. A further finding was that microcapsules showed the highest entrapment (7.5%) and encapsulation efficiency (98.5%).	Mehyar et al. (2014)
Curcumin	Hydrolyzed GG	Encapsulation of curcumin covering with hydrolyzed GG resulted in increased mechanical and physicochemical stability for weeks.	Manna et al. (2015)

##### Encapsulation

**FIGURE 5:**
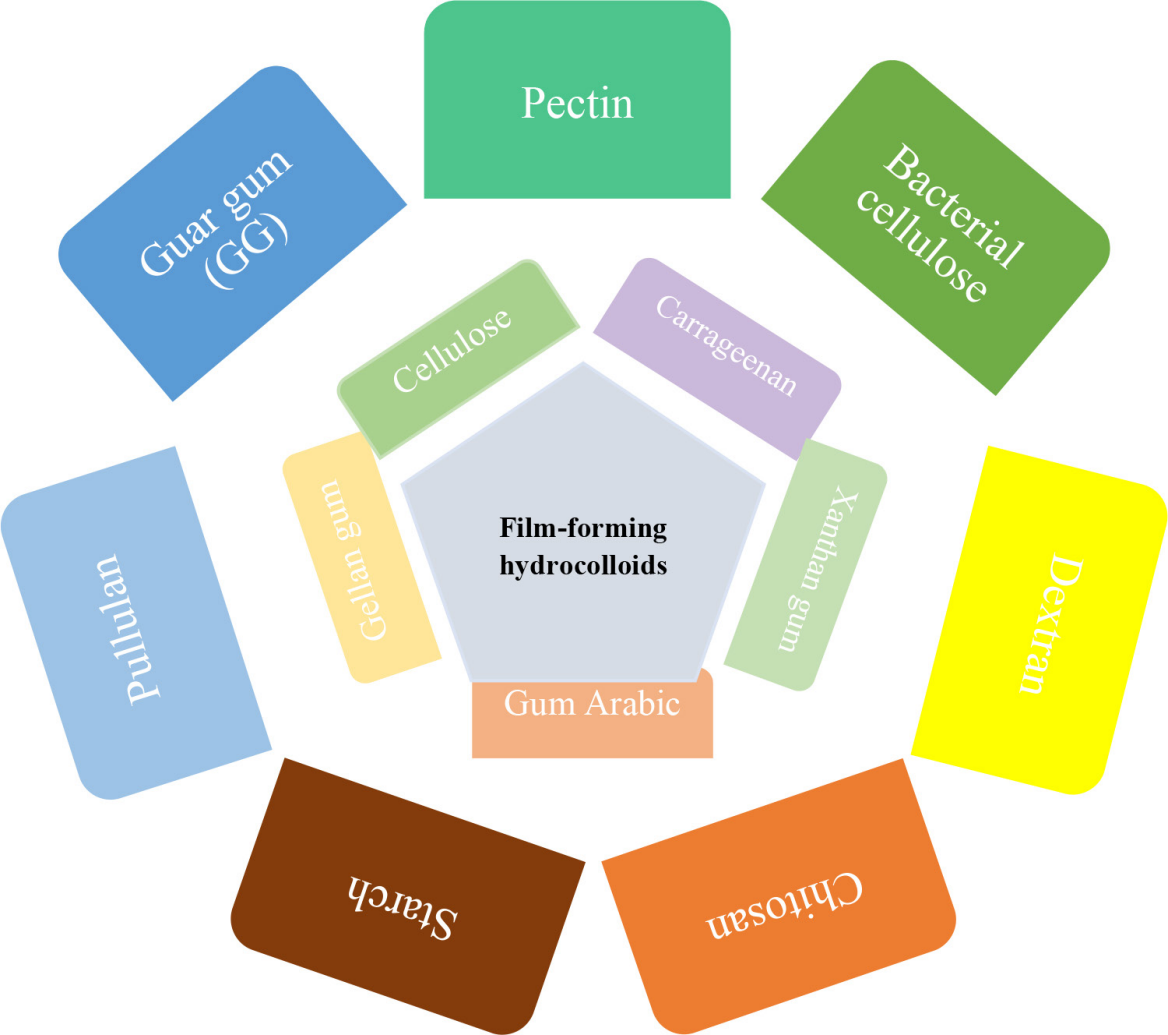
Hydrocolloids employed in encapsulation technique.

An encapsulation process involves encasing active ingredients or other core materials inside a wall for protection or to be released later. The main advantage of using different hydrocolloids (Figure 5) as the outer shell of encapsulates is their edible, biodegradable nature and ability to form a barrier between the core and the environment surrounding it (Duran et al., 1994; Nedovic et al., 2011). In addition to alginate‐coated gelatin microsphere encapsulation, alginate–starch encapsulation, whey protein gel encapsulation, and others, hydrocolloid encapsulation systems are available (Vidhyalakshmi et al., 2009). This can be achieved by including complexation, thermal gelation, fluidized bed coating, coacervation, interfacial emulsion polymerization, extrusion coating, ionic gelation, lyophilization, liposomal encapsulation, crystallization, and spray drying among others (Vemmer & Patel, 2013).

Several reasons can be attributed to the use of encapsulation technology (Bratovcic & Suljagic, 2019; Chatzidaki et al., 2019). A significant advantage of this technology is the possibility of protecting sensitive food ingredients (ascorbic acid or anthocyanin) and bioactive compounds (probiotics and prebiotics) from being inactivated (Vemmer & Patel, 2013). For instance, GG and cationic guar gum (CGG) liposomes were prepared to improve phospholipid bilayer membrane stability and reduce curcumin degradation by Pu et al. (2019). A transmission electron microscopy confirmed the presence of GG and CGG coated on the surface of the vesicles; furthermore, dynamic light scattering, atomic force microscopy, and Fourier transform infrared spectroscopy analysis confirmed that both layers of GG and CGG were successfully coated on the surface of the vesicles. It was found that CGG was more effective at stabilizing structures than GG by decreasing membrane fluidity and enhancing the lateral packing of lipids. A further examination of the stability of the liposome coating, particularly the modified coating with CGG on the surface, led to the conclusion that it could effectively protect curcumin from the damaging effects of oxidation or heat (Pu et al., 2019). In conclusion, GG and its derivatives could be developed to encapsulate and deliver functions in the food industry (Table 3).

Moreover, Toniazzo et al. (2014) produced liposomes loaded with β‐carotene and tested their incorporation with yogurt to test their efficacy. After 95 days, nearly 90% of the encapsulated carotenoids were retained, suggesting that liposomes can shield β‐carotene from degradation. Based on the thermal analyses, the liposome bilayer structure was unaffected by either the β‐carotene or gums (xanthan gum and GG). As a result, the gum mixture proved highly effective in preventing the aggregation of β‐carotene‐loaded liposomes during storage, indicating that it is possible to develop a functional yogurt with β‐carotene encapsulated in liposomes (Toniazzo et al., 2014).

On the other hand, probiotics contain live microorganisms, either monocultures or mixed cultures, that confer health benefits to their hosts when administered in sufficient amounts. Probiotics are derived mainly from species of *Bifidobacterium* and *Lactobacillus* (Ozyurt & Ötles, 2014). It is primarily related to improving the health of the digestive system and preventing diseases associated with this organ. Temperature, oxygen, acidity, and bile salt solutions are factors that they are susceptible to. In this way, it is difficult for free probiotics to maintain viability during processing or storage when they are not covered (Ding & Shah, 2009; Reque & Brandelli, 2021). As an alternative method for protecting sensitive compounds, microencapsulation has evolved into an effective way to cultivate and protect the encapsulated microorganisms as well as enhance their viability by providing a specific microenvironment (de Barros Fernandes et al., 2014). Based on the study of Ainsley Reid et al. (2007), they revealed that probiotic‐loaded microcapsules with alginate and also GG covering had the potential to generate several health benefits, and they were more stable than capsules with whey protein covering during 32 days of storage (Reid et al., 2007).

Furthermore, microencapsulation could also benefit the food industry as a means of controlling release, masking unpleasant tastes, and immobilizing cells or food enzymes in food processing, to name a few. While the overall benefits outweigh the negatives, the following potential drawbacks such as additional costs, increased complexity of processing and supply chains, undesirable consumer notice of encapsulates in food products, and challenges with stability during processing and storage must be overcome (Zuidam & Nedovic, 2010). An experiment was designed by Guarienti et al. (2021) to microencapsulate *Spirulina* (as a food supplement), using alginate/GG with the ionic gelation method, with controlled release in various pHs and then to assess the bioactivity (stability and antioxidant activity) of microencapsulated *Spirulina* to formulate new food products and increase their industrial use. Findings showed that the antioxidative activity of *Spirulina* was preserved during adverse pHs (like 1.2), and the microencapsulation matrix prevented denaturation at high temperatures (100°C). As a result, *Spirulina* biomass can be inserted into food matrices without losing its properties, followed by *Spirulina* microcapsules that can be produced in an alternative manner based on the technique and conditions used (Guarienti et al., 2021).

There is also a second reason for encapsulating products, such as essential oils and aromas, to prevent their loss and degradation by evaporation. The coacervation of proteins with hydrocolloid complexes is commonly used as a method of flavor encapsulation (Duran et al., 1994). The protein and some hydrocolloids are combined at a pH below the protein isoelectric point in order to allow the protein and the hydrocolloid to form a complex, which precipitates the flavor materials at the coacervate stage (Koupantsis et al., 2014). In the formulation of encapsulation, carboxymethyl cellulose or GG was found to reduce the volatility of highly volatile non‐polar flavor compounds such as α‐pinene, ethyl 2‐methylbutyrate, and 1,8‐cineole, while components with less volatile properties such as vanillin, maltol, and methyl anthranilate did not (Buljeta et al., 2021; Preininger, 2006). For instance, GG‐based encapsulation of mint oil using spray drying improved the quality and bioactivity during storage time (Sarkar et al., 2013).

The main limitation of GG is that it forms highly viscous solutions. For this reason, partial hydrolysis is used to modify and depolymerize GG, including acids, enzymes, heat, and ultrasonication, to achieve low molar masses (Chadha, 2021). Kuck and Noreña (2016) found that spray‐dried microcapsules of grape skin phenolic extract with gum arabic and PHGG as wall materials had better physicochemical properties than free extract (Kuck & Noreña, 2016). Another encapsulation strategy works very well in terms of encapsulating curcumin using conjugation. Encapsulation of curcumin covered with hydrolyzed GG increased mechanical and physicochemical stability over weeks (Manna et al., 2015).

## 5 CONCLUSION AND FUTURE PROSPECTIVE

This material can be explored further in other fields as an important agrochemical derived from the seed endosperm of the guar plant *Cyamopsis tetragonolobus*. This compound strongly forms hydrogen bonds in water, making it a useful thickener and stabilizer. It is important to keep in mind that guar gum aqueous solutions are very viscous. This is why it has a wide variety of applications in a wide range of industries like food and pharmaceuticals. Besides the fact that it is used in many industries, its popularity has also been attributed to its low cost. Firstly, its economic nature makes it popular in the gums and stabilizers industry, which can be found in ice cream, sauces, beverages, bakeries, and meat industries. Additionally, it is used in food products in the form of dietary fiber as a supplement. Therefore, the rising demand for gluten‐free and plant‐based food products has created new opportunities for guar gum in the food industry. Guar gum is a gluten‐free and vegan‐friendly alternative to other thickening agents, making it an ideal choice for health‐conscious consumers. Secondly, its consumption reduces the risk of heart disease by lowering the cholesterol level in the body and controlling diabetes in human beings. Thirdly, the increasing use of guar gum in functional food products is expected to drive its growth in the future. GG is a prebiotic fiber that promotes gut health by stimulating the growth of beneficial gut bacteria. The use of guar gum in functional food products such as probiotic yogurts, energy bars, and dietary supplements is expected to increase in the future. This will create new opportunities for guar gum in the food industry, especially in the health and wellness segment. Therefore, the development of sustainable sourcing practices and the use of alternative thickeners can help ensure the availability and affordability of guar gum in the food industry.

## AUTHOR CONTRIBUTIONS

**Sima Tahmouzi:** Data curation (equal); resources (equal); writing – original draft (equal). **Saba Eyshi:** Methodology (equal); resources (equal); visualization (equal). **Amin Mahmoudzadeh:** Investigation (equal); resources (equal); writing – original draft (equal). **Behnam Alizadeh:** Investigation (equal); resources (equal); visualization (equal); writing – original draft (equal). **Heidar Meftahizade:** Conceptualization (equal); investigation (equal); project administration (equal); supervision (equal); writing – original draft (equal). **Neda Mollakhalili‐Meybodi:** Resources (equal); validation (equal); writing – original draft (equal). **Mehrnaz Hatami:** Investigation (equal); resources (equal); writing – review and editing (equal).

## CONFLICT OF INTEREST STATEMENT

The authors declare that they have no conflict of interest.

## Data Availability

The data that support the findings of this study are available on request from the corresponding author.
